# Identification and validation of immune-associated NETosis subtypes and biomarkers in anti-neutrophil cytoplasmic antibody associated glomerulonephritis

**DOI:** 10.3389/fimmu.2023.1177968

**Published:** 2023-07-03

**Authors:** Mi Tao, Yiqing He, Lijuan Li, Yuyan Li, Wenwen Liao, Haihang Nie, Ping Gao

**Affiliations:** ^1^ Department of Nephrology, Zhongnan Hospital, Wuhan University, Wuhan, China; ^2^ Department of Hematology, Zhongnan Hospital, Wuhan University, Wuhan, China; ^3^ Department of Gastroenterology, Zhongnan Hospital, Wuhan University, Wuhan, China

**Keywords:** ANCA-associated vasculitis, glomerulonephritis, NETosis, immune characteristics, bioinformatics

## Abstract

**Background:**

NETosis is a new form of cell death, marked by DNA chromatin release from dead neutrophils. While it aids in microbe defense, it may worsen inflammation in autoimmune diseases, causing tissue harm. The impact of NETosis on Anti-neutrophil Cytoplasmic Antibody-associated Glomerulonephritis (ANCA-GN) remains unexplored and requires investigation.

**Methods:**

First, a weighted gene co-expression network analysis (WGCNA) was conducted to uncover differential expression of neutrophil extranuclear trap-associated genes (DE-NETs) in ANCA-GN. The NETosisScore model was established through the single sample gene set enrichment analysis (ssGSEA), which categorized all patients into high-risk and low-risk groups. The accuracy of model was assessed by ROC curve. The biological function of various subgroups was explored through Gene Set Variation Analysis (GSVA), while the abundance of immune cell infiltration was measured with CIBERSORT. Furthermore, the key NETosis-related genes (NRGs) were identified using three machine learning algorithms, and their relationship with renal function was analyzed through the NephroseqV5 database. Through the application of qPCR and immunohistochemical staining techniques, the mRNA and protein expression levels of NRGs were determined in patients with ANCA-GN and control.

**Results:**

A NETosisScore model was developed from 18 DE-NETs using the ssGSEA algorithm. The model’s ability to predict ANCA-GN patients with a ROC AUC of 0.921. The high-risk group in ANCA-GN showed enrichment of immune-related pathways and greater infiltration of immune cells, as revealed by KEGG enrichment analysis and CIBERSORT. Using three machine learning algorithms, we identified six NRGs. Significant positive correlations were found between NRGs and CCR, macrophages, T-cell co-inhibition, and TIL. Further KEGG analysis revealed that the functions of NRGs may be closely related to the toll-like receptor signaling pathway. The levels of NRGs increased as kidney function declined and were positively correlated with Scr (serum creatinine) and negatively correlated with GFR (glomerular filtration rate), qPCR analysis showed increased expression of most NRGs in ANCA-GN patients. Furthermore, immunohistochemical staining confirmed higher expression of all NRGs in ANCA-GN patients.

**Conclusion:**

NETosisScore model accurately predicts high-risk patients in ANCA-GN with enriched immune pathways, 6 NRGs identified as potential biomarkers.

## Introduction

As a major subgroup of vasculitis, antineutrophil cytoplasmic antibody associated vasculitis (AAV) is a relatively rare group of autoimmune diseases characterized by its effect on small blood vessels. AAV is particularly prone to renal involvement, which is usually characterized by quickly progressing glomerulonephritis with renal failure, hematuria, and glomerulonephritis (1). Glomerulonephritis caused by AAV is called ANCA-associated glomerulonephritis (ANCA-GN), which can lead to acute kidney injury (AKI), chronic kidney disease (CKD), end-stage renal disease (ESRD), and even death (2).At present, although traditional therapy, including hormones and immunosuppressants, can significantly improve the prognosis of patients, the mortality of AAV patients is still 2 to 3 times higher than that of the general population matched by age and sex (3).At the same time, the side effect of immunosuppressive therapy is still the main cause of early death of AAV (4). In recent years, a list of drugs that target AAV, including C5/C5a receptor antagonists of complement replacement pathway, Abatacepta and Alemtuzumab for T lymphocytes and Belimumab for B lymphocytes, have been developed rapidly because of their less toxicity and more individuation (5–9). However, most drugs still require extensive human trials. At the same time, the initial nonspecific and heterogeneous presentation of ANCA-GN may delay the diagnosis and treatment. Therefore, it is of great significance to explore the pathogenesis of ANCA-GN and find new targets for early diagnosis and intervention.

NETs (neutrophil extranuclear traps) is a network of densified chromatin, histones, cytoplasmic and granular proteins in the mitochondria of the neutrophil nucleus (10). NETs not only plays a key role in the pathogenesis of some metabolic diseases (11), autoinflammatory diseases (12) and septicemia (13), but also closely related to AAV (14). Studies *in vitro* and *in vivo* have shown that NETs may be associated with disease-related thrombosis (15), direct toxicity of endothelial cells (16) and vascular injury (17) in patients with AAV. It may be due to the endothelial damage caused by NETs produced by neutrophils stimulated by ANCA, which activates the alternative complement pathway, further expands the inflammatory process, and ultimately, forms a vicious circle of neutrophil recruitment and activation. Therefore, NETs may be involved in the occurrence and development of AAV. It is worth noting that when nuclear chromatids in AAV are squeezed out of the outer space of cells as NETs, it can induce a new kind of cell death, called NETosis (18). Victoria et al. found that acting on NETosis in different ways may reduce the severity of many diseases and thus improve survival (19). However, there are few studies on NETosis in ANCA-GN. Therefore, this study mainly discusses the role of NETosis in ANCA-GN and its relationship with immune microenvironment, which may provide a new direction for the treatment of ANCA-GN.

In the study, weighted gene coexpression network analysis (WGCNA) was used to identify modules related to clinical traits in ANCA-GN, intersected with NETs-related genes and identified differentially expressed NETs (DE-NETs). DE-NETs are used to build the NETosisScore model, which was used to divide samples into high- and low-risk groups. The gene expression, immune microenvironment, and biological function of these groups were explored. NETosis-related genes (NRGs) were screened by three machine learning methods and explored for their potential biological function, immune cell invasion, and clinical value. Differential expression of most NRGs was verified by QPCR in whole blood samples from patients with ANCA-GN, providing new insights for intervention.

## Methods

### Data download and preparation

**Figure 1:**
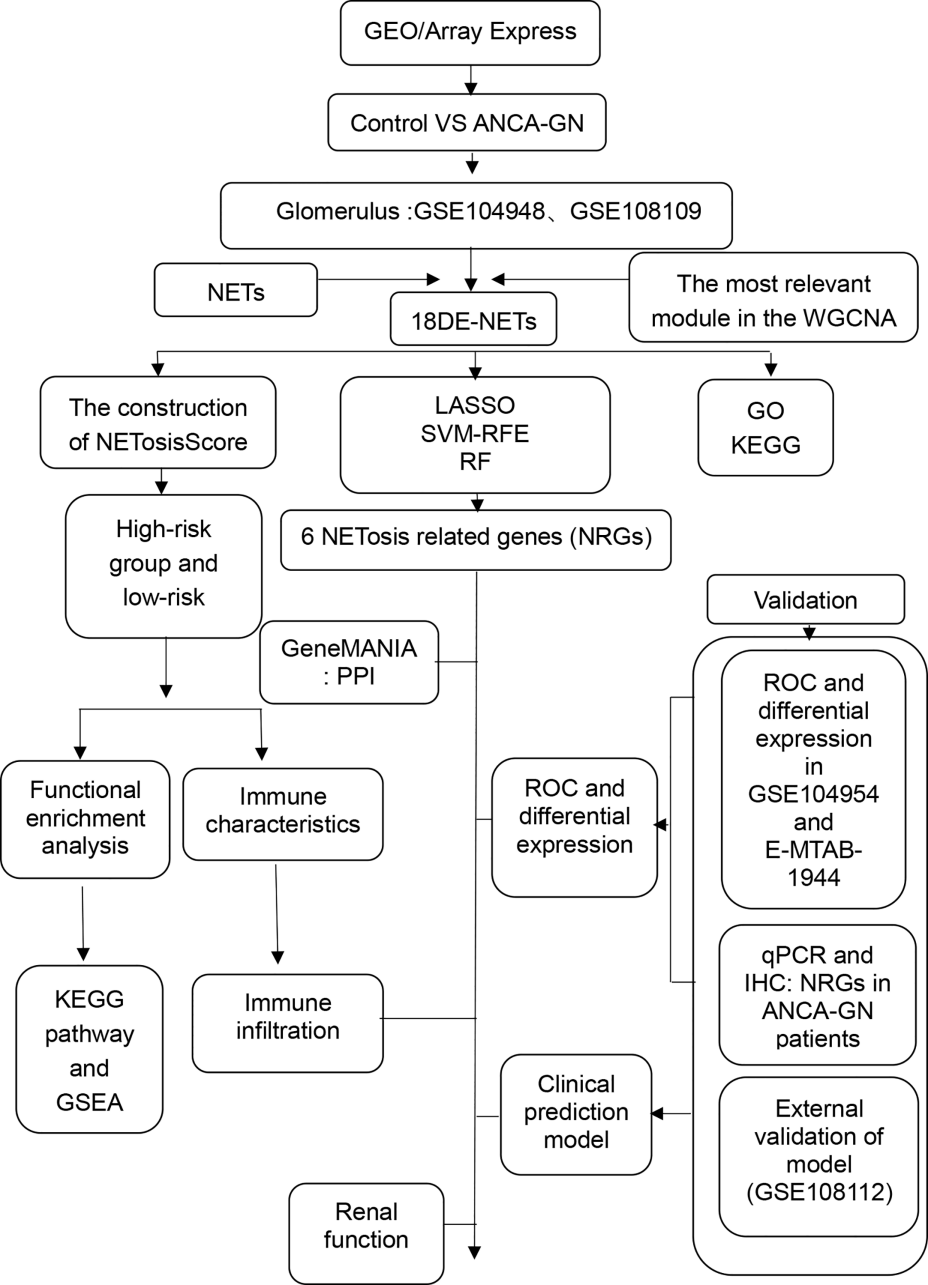
Study flow chart. ANCA-GN, anti-neutrophil cytoplasmic antibody associated glomerulonephritis.DE-NETs, differentially expressed neutrophil extranuclear traps related genes.

The study flow chart is shown in **Figure 1**. Five primary datasets (GSE104954, GSE108112, GSE104948 GSE108109, E-MTAB-1944) were downloaded from GEO database (https://www.ncbi.nlm.nih.gov/geo/) and the Array Express database (https://www.ebi.ac.uk/arrayexpress/). Information about these data sets can be found in **Supplementary Table 1**. Gene sets associated with NETs were derived from previous studies (20). GSE108109 and GSE104948 are merged into a data set as a training set, while GSE104954, GSE108112 and E-MTAB-1944 are all used as independent verification sets. The “Combat” algorithm is used to eliminate the batch effect (**Supplementary Figures 1A-E**).

### Weighted correlation network analysis in ANCA-GN

First, the top 25% of genes with low expression in these datasets were deleted. Weighted correlation network analysis (WGCNA) was used to construct co-expressed networks and identify co-expression modules. Based on the scale-free topology criterion, an appropriate soft-thresholding power (β) was chosen by the pick Soft threshold plugin in R software. Then, to identify various modules, hierarchical clustering and the dynamic tree cut function were applied. Finally, the module which was most related to the clinical traits of ANCA-GN was screened out, and the genes in the module was identified as potential biomarkers of ANCA-GN. Differentially expressed NETs (DE-NETs) in ANCA-GN were obtained by intercrossing the most significant module genes with NETs.

### Functional enrichment analysis of DE-NETs

Using the R ‘clusterProfiler’ and ‘enrichment plot’ packages, Gene Ontology (GO) and Kyoto Encyclopedia of Genes and Genomes (KEGG) of DE-NETs were carried out.

### The construction and validation of NETosisScore model

Based on the results of WGCNA and differential expression, 18 DE-NETs from two glomerular sample cohorts (GSE108109 and GSE104948) were included in subsequent analyses, including CYBB, IL1B, IL6, ITGAM, ITGB2, and MMP9, PTAFR, TLR2, TLR7, TLR8, BST1, CD93, CSF3R, FCGR3B, FPR1, HPSE, LILRB2 and PDE4B. Then, the DE-NETs of each sample in all cohorts was quantitatively analyzed by single sample gene set enrichment analysis (ssGSEA), and the NETosisScore was constructed by ssGSEA score. According to the median ssGSEA score, patients were divided into two groups: low risk group and high-risk group. The above operation is done by the “GAVA” R package. Principal component analysis (PCA) was used to further verify the expression of DE-NETs among different NETosisScore subgroups, and the prediction ability of NETosisScore was quantified by the area under ROC curve. Grouping information can be found in **Supplement Table 2**

### Immunoinfiltration and biological function between NETosisScore subgroups

Based on the gene sets of 29 immune cell infiltration in previous studies (21), the immune cell infiltration in the high and low risk group of NETosisScore was quantitatively analyzed by ssGSEA.

The GSVA technique and the ‘limma’ R package was used to compare the route activation scores between the NETosisScore subgroups. From the MSigDB database, the gene sets “c2.cp.kegg. v7.0. symbols” was downloaded for GSVA analysis. Analysis of variance was set to adjust for *P*< 0.01 as the cutoff value. Enrichment analysis was performed for high-risk and low-risk groups in NETosisScore using the R package “clusterProfiler”. At the same time, Gene set enrichment analysis (GSEA) was used to further describe the biological function between high-risk and low-risk groups of NETosisScore. *p* < 0.05; FDR<0.25 was statistically significant.

### Identification of NETosis-related genes

Based on DE-NETs, NRGs were identified by three machine learning algorithms LASSO regression (22), SVM-RFE (23) and Random forest algorithm (24). NRGs was confirmed using the independent dataset GSE104954 and E-MTAB-1944, and their diagnostic power was evaluated with the area under the ROC curve utilizing the independent dataset GSE104954 and E-MTAB-1944.

### Construction of protein-protein interaction for NRGs

The tool GeneMANIA (http://genemania.org/) is frequently used to build PPI networks (25). To locate additional genes connected to a group of input genes, a lot of functional association data is used. While pathways, co-expression, co-localization, and protein domain similarity are all included in association data along with protein and genetic connections. GeneMANIA can be used to discover new members of a pathway or complex, searching for additional genes that could have escaped the screen, as well as to find novel genes with particular roles, such as protein kinases.

### Construction and validation of clinical diagnostic models

The combined data were used as the training set, and the “rms” package was used to construct a diagnostic nomogram based on NRGs. The model’s capacity for discriminating was evaluated using the area under the ROC curve. The model’s calibration was assessed using the calibration curve. The clinical benefit of the model was measured using clinical impact curve (CIC) and decision curve analysis (DCA). GSE104954 and GSE108112 were used as independent sets to validate the calibration and diagnostic efficacy of the model, respectively.

### Correlation analysis of NRGs and immune characteristics

ssGSEA defines an enrichment score to show the absolute enrichment of a particular gene set in each sample and is used to assess the quantity of specific infiltrating immune cells (26). The list of genes in the infiltrating immune cell gene set was derived from previous studies.

### Renal function correlation with NRGs

The NephroseqV5 tool is used to determine the correlation between NRGs and renal function. We downloaded the expression data of NRGs and used “ggplot2” to redraw the scatter chart.

### Recruitment of ANCA-GN patients and collection of clinical ANCA-GN samples

In an observational study conducted at Zhongnan Hospital of Wuhan University, three renal biopsy specimens from ANCA-GN patients were obtained from the Department of Nephrology, alongside four whole blood samples from ANCA-GN patients. Age and gender-matched whole blood samples were collected from healthy individuals at the Physical Examination Center. Additionally, three peritumoral tissue samples were acquired from patients diagnosed with renal clear cell carcinoma who had undergone nephrectomy at the Department of Urology. This study was approved by the Ethics Committee of Zhongna Hospital, Wuhan University (2022122K) and adhered to the Declaration of Helsinki and the CONSORT guidelines.

The study population comprised four groups: 1) Normal peritumoral renal tissue from patients aged ≥18 years with primary renal cancer who underwent nephrectomy. Patients with multiple metastases, other types of cancer, or insufficient clinical data were excluded. Renal cancer samples were collected immediately after resection in the operating room and stored at -80°C. All specimens were pathologically confirmed as ccRCC. 2) Renal tissue and 3) Whole blood samples from ANCA-GN patients: Inclusion criteria were a confirmed ANCA-GN diagnosis based on clinical presentation, laboratory results, and kidney biopsy; age >18 years; no immunosuppressive therapy or sample collection at least four weeks after stopping treatment. Exclusion criteria included other types of kidney disease or autoimmune disorders, severe organ dysfunction, and pregnancy, lactation, or planning to conceive. 4) Healthy control participants aged >18 years, matched for age and sex with ANCA-GN patients, were required to have normal physical examination results and no history of kidney disease, autoimmune disorders, or other chronic conditions. Exclusion criteria included chronic diseases, acute infections, recent surgery, pregnancy, lactation, planning to conceive, or family history of kidney disease, autoimmune disorders, or other hereditary diseases. Detailed patient information is provided in **Supplementary Table 3**.

### qPCR

After separating the blood cells in each sample using a centrifuge, the plasma fraction was collected and kept at -80°C until it was needed. Informed consent was acquired from patients who were exempt before the study utilized the remaining samples. Measurement and isolation of RNA Using Trizol reagent from Invitrogen, Carlsbad, California, USA, total RNA was isolated from the venous blood of patients with ANCA-associated vasculitis and control. Prime Script RT kit and total RNA were used to create complementary DNA (cDNA) (Takara). On a CFX-96 equipment, SYBR-Green assay was utilized for qPCR (Bio-Rad Laboratories, Inc, USA). GAPDH was used to standardize the data after being computed using the 2^-△△Ct^ method. Primer sequences of all NRGs used for qPCR in this study are listed in **Supplementary Table 4**.

### Immunohistochemical staining

Three ANCA-GN patients and three age and sex-matched ccRCC patients’ adjacent non-cancerous renal tissues were collected from the Department of Nephrology and the Department of Urology of Zhongnan Hospital of Wuhan University, respectively. The slides were observed and imaged using a light microscope. To ascertain the protein expression levels of NRGs in ANCA-GN and healthy control samples, immunohistochemical (IHC) staining was performed for CYBB (Proteintech, Rabbit: 19013-1-AP), ITGAM (Affinity, Rabbit: DF2911), ITGB2 (Proteintech, Rabbit: 10554-1-AP), TLR2 (Proteintech, Rabbit: 17236-1-AP), LILRB2 (ABclonal, Rabbit: A10135) and TLR7 (abcam, Rabbit: ab124928).The tissue samples were fixed in 4% paraformaldehyde and embedded in paraffin. After sectioning at a thickness of 2 µm, the sections were deparaffinized in xylene, rehydrated in graded alcohol, and rinsed with phosphate-buffered saline (PBS). Antigen retrieval was performed using sodium citrate buffer (pH 6.0) at 95°C for 15 min. Endogenous peroxidase activity was blocked by incubation with 3% hydrogen peroxide for 10 min. The sections were then incubated with primary antibodies against the target proteins (diluted 1:100) overnight at 4°C. The next day, the sections were washed with PBS and incubated with secondary antibodies for 1 h at room temperature. After washing again with PBS, the sections were incubated with horseradish peroxidase-conjugated streptavidin for 30 min. Finally, the sections were visualized using 3,3’-diaminobenzidine (DAB) and counterstained with hematoxylin. Negative controls were treated with PBS instead of primary antibodies.

### Statistical analysis

Student t test and Wilcox test were used for differential expression and enrichment scores of immune cell abundance in experimental group and control group, respectively. Kruskal test was used to evaluate gene expression and infiltrated immune cell abundance between high-risk and low-risk groups in NETosisScore. Spearman correlation was used to analyze the correlation between NRGs and immune cell invasion, GFR and Scr. All statistical analyses were performed using R (v.4.2.2) software. *P <*0.05 was considered statistically significant.

## Results

### Co-expression network construction and hub module identification

**Figure 2:**
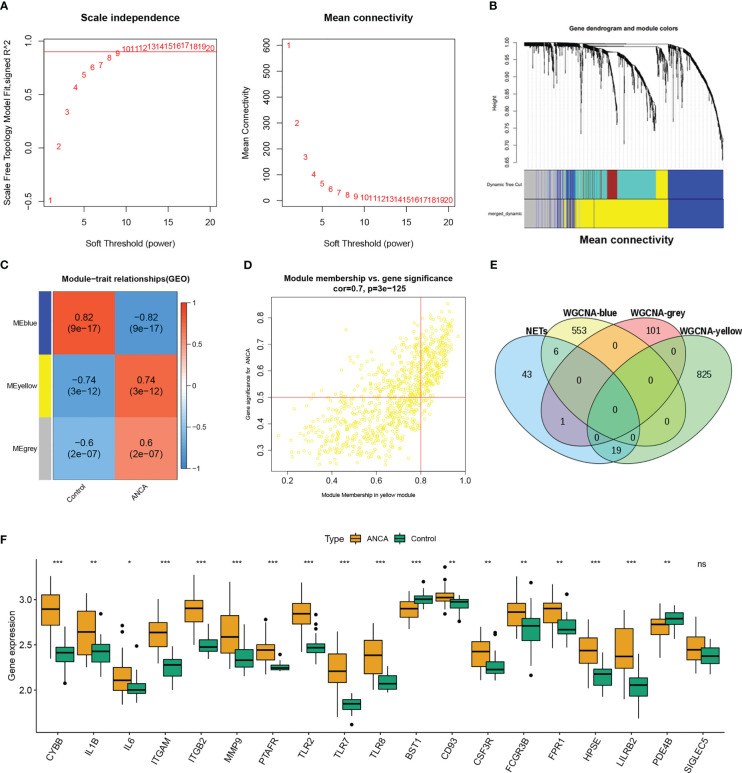
Construction of WGCNA modules. **(A)** Soft threshold β*=*9 and scale–free topological fit index (R2). **(B)** Shows the original and combined modules under the clustering tree. **(C)** Heatmap of the module-trait relationships. **(D)** Scatter plot for correlation between gene module membership in the yellow module and gene significance. **(E)** Venn diagram of WGCNA module genes and NETs. **(F)** Box plot shows the differential expression of 18 DE-NETs between healthy control and ANCA-GN. WGCNA, weighted gene co-expression network analysis; ANCA-GN, anti-neutrophil cytoplasmic antibody associated glomerulonephritis; DE-NETs, differentially expressed neutrophil extranuclear traps related genes. *p < 0.05; **p < 0.01; ***p < 0.001; ns, no significant difference.

To screen out the differentially expressed genes in ANCA-GN. First, after correcting for batch effects, the two datasets GSE108109 and GSE104948 were combined into a single cohort for further analysis. Next, WGCNA analysis was used to search for co-expressed gene modules in ANCA-GN. After excluding genes with mean expression less than 1 and retaining the top 25% of genes with the largest variance, the 27 control samples and 37 AAV samples were clustered. To be coincident with the scale-free network, a soft threshold of β = 9 (scale-free R2 = 0.9) was chosen (**Figure 2A**). The strongly correlated modules are merged using a clustering height threshold of 0.25 and shown below the clustering tree (**Figure 2B**). Three modules related to ANCA-GN were identified (**Figure 2C**). To filter out the modules most associated with NETosis, genes from these three modules were intersected with the reported NETs gene set, and Wayne plots revealed that the yellow module had the most intersections with NETs (**Figure 2E**). By calculating correlations between the modules and clinical characteristics, it is found that there is a significant positive correlation between the yellow module and ANCA-GN group (r=0.79, p=3e-12). In addition, a significant correlation was also found between GS and MM based on scatter plots in the yellow module (**Figure 2D**). Therefore, the yellow module was identified as the most relevant module for NETosis with ANCA-GN. As can be seen from (**Figure 2F**), there was no difference in SIGLEC5 expression between ANCA-GN and control group, finally,18 DE-NETs were included in the study.

### Functional analysis of DE-NETs

**Figure 3:**
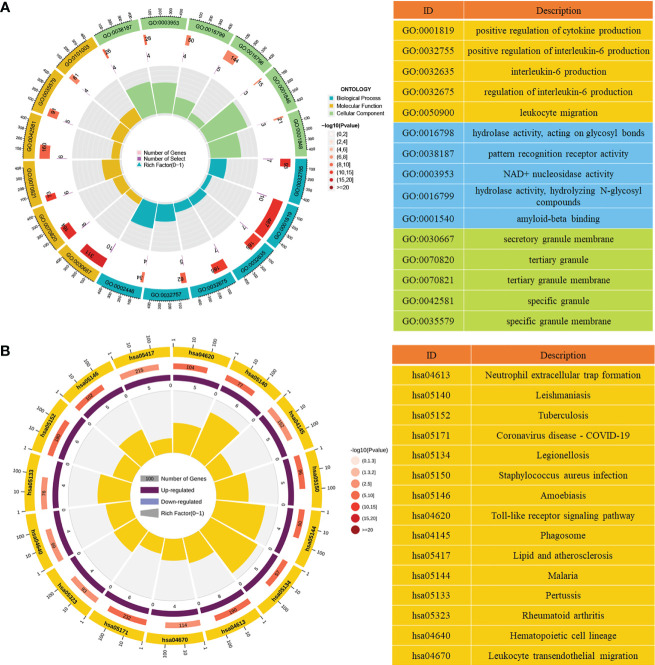
Functional analysis of DE-NETs. **(A)** GO enrichment analysis of DE-NETs, the first circle represents the top 15 GO entries, and the number of genes corresponds to the outer circle. The second circle represents the number of genes in the genome and the p-value of DE-NETs enrichment in a specific biological process. The third circle represents the enrichment factors of GO terms. **(B)** KEGG enrichment analysis of DE-NETs, the first circle represents the number of the top 15 KEGG terms and genes corresponding to the outer ring; The second circle represents the number of genes in the genome and the P value of enrichment. The third circle indicates the ratio of up-regulated genes (dark purple) to down-regulated genes (light purple). The fourth circle represents the enrichment factors of KEGG. GO, Gene ontology; KEGG, Kyoto Encyclopedia of Genes and Genomes; DE-NETs, differentially expressed neutrophil extranuclear traps related genes.

To clarify the potential biological significance of DE-NETs in ANCA-GN, both GO enrichment analysis and KEGG enrichment analysis were performed. In the GO enrichment analysis, “positive regulation of interleukin-6 production”, “positive regulation of cytokine production” and “positive regulation of interleukin-8 production” were mainly enriched in biological processes (BP), “secretory granule membrane”, tertiary granule” and “ficolin−1−rich granule” are mainly enriched in cell composition (CC), while “hydrolase activity, acting on glycosyl bonds”, “pattern recognition receptor activity” and “NAD+ nucleosidase activity” are mainly enriched in molecular function (MF) (**Figure 3A**; **Supplementary Table 5**). In the KEGG enrichment analysis, “neutrophil extracellular trap formation”, “toll-like receptor signaling pathway” and “staphylococcus aureus infection” were predominantly enriched (**Figure 3B**; **Supplementary Table 6**), suggesting that inflammatory factor production and toll-like receptor signaling pathways may be involved in the formation of NETosis in ANCA-GN.

### Construction and validation of NETosisScore model

**Figure 4:**
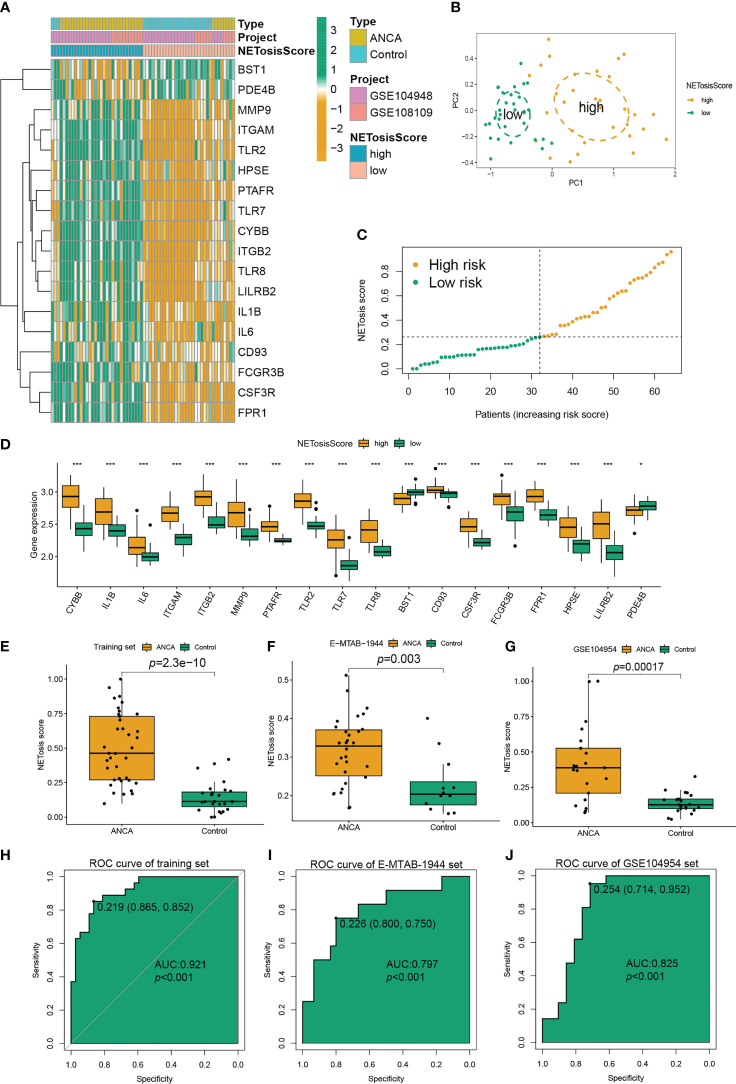
Construction and verification of the NETosisScore model in ANCA-GN. **(A)** Expression profiles of the high-risk group and low-risk group of NETosisScore were constructed based on two cohorts (GSE104948 and GSE108109). **(B)** PCA between the high-risk and low-risk groups. **(C)** Boxplot showing the distribution of NETosisScore in healthy and ANCA-GN samples. **(D)** Boxplot illustrating the differences in expression levels of 18 DE-NETs between the high-risk and low-risk groups. **(E)** Boxplot of the distribution of NETosisScore in the training set. **(F, G)** ROC curves generated by the NETosisScore model in the validation set (E-MTAB-1994 and GSE104954). **(H)** Boxplot of the distribution of NETosisScore in the training set. **(I, J)** ROC curves generated by the NETosisScore model in the validation set (E-MTAB-1994 and GSE104954). ANCA-GN, anti-neutrophil cytoplasmic antibody-associated glomerulonephritis; PCA, Principal Component Analysis. *p < 0.05; ***p < 0.001.

To confirm the expression pattern of NETosis in ANCA-GN, 64 patients from two ANCA-GN cohorts (GSE104948 and GSE108109) were included in the metacohort as a training set for further analysis. A NETosis Score model (NETosisScore) was constructed based on the median ssGSEA score of 18DE-NETs, and 64 patients were divided into high-risk and low-risk groups (**Figure 4A**). The distributions between the high-risk and low-risk groups could be clearly distinguished by principal component analysis (PCA) (**Figure 4B**). As can be seen from the NETosisScore distribution maps of the two groups, with the increase of risk score, the number of patients in the high-risk group gradually increased (**Figure 4C**). Meanwhile, all DE-NETs were significantly differentially expressed between the high-risk and low-risk groups (**Figure 4D**). The training set and two independent validation sets (E-MTAB-1994 and GSE104954) were included to investigate the ability of the NETosisScore model to discriminate between ANCA-GN patients and controls. In the training set, as shown in the box plot, NETosisScore could significantly discriminate between normal controls and the ANCA-GN patients (p=2.3e-10), and NETosisScore was higher in the ANCA-GN patients compared to normal controls (**Figure 4E**); the area under the ROC curve of NETosisScore in the training set was 0.920 (p<0.001) (**Figure 4H**). Further, the discriminative power of the NETosisScore model was validated by independent datasets. Not only was the NETosisScore of the ANCA-GN group statistically significantly higher than the control group in both the E-MTAB-1994 set (p=0.003) (**Figure 4F**) and the GSE104954 set (p=0.00017) (**Figure 4G**), but the area of the ROC curve was found to be 0.797 in the E-MTAB-1994 set (p< 0.001) (**Figure 4I**), and 0.825 (p<0.001) for the GSE104954 set (**Figure 4J**),suggesting that the NETosisScore model has strong diagnostic power and that ANCA-GN patients may have different patterns of NETosis expression.

### Immunological characteristics and biological pathways of two NETosisScore subtypes

**Figure 5:**
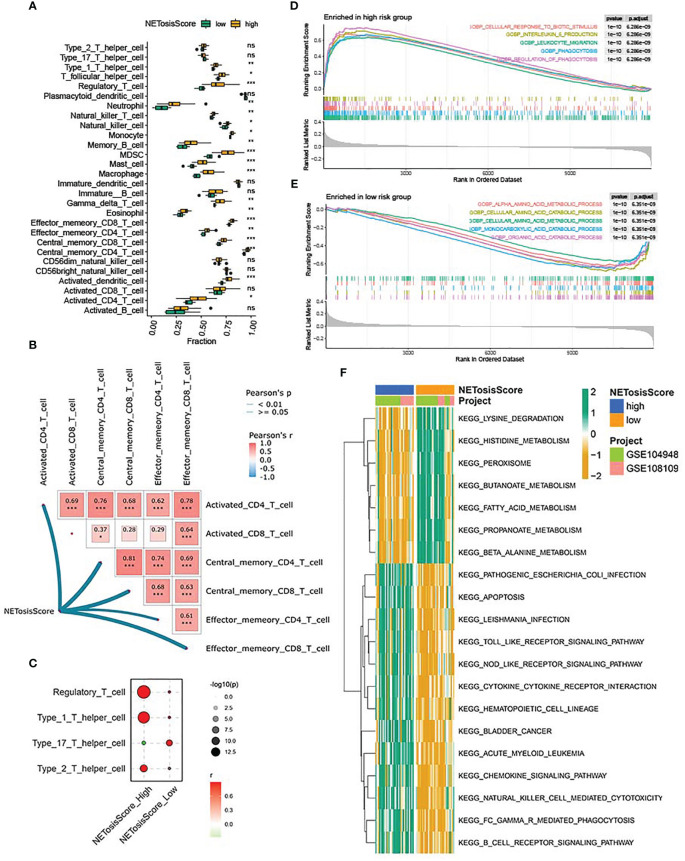
Immune characteristics and functional enrichment analysis of NETosisScore subtypes. **(A)** Abundance differences of 28 infiltrating immune cells between NETosisScore subgroups. **(B)** NETosisScore correlation with CD4T cells and CD8T cells network heat map. **(C)** Correlation Bubble Plot of NETosisScore Groups with CD4 T Cell Subsets. **(D, E)** GSEA enrichment analysis of NETosisScore subtype in biological processes. **(F)** Differential enrichment of KEGG pathways among NETosisScore subtypes. *p < 0.05; **p < 0.01; ***p < 0.001; ns, no significant difference.

To further determine the immune characteristics between high-risk and low-risk groups, the abundance of immune cell infiltration was compared between high - and low-risk groups by the CIBERSORT algorithm. The result showed that, compared with the low-risk group, the abundance of immune cell infiltration was higher in the high-risk group. It is worth noting that activated CD4 T cells, central CD4 memory T cells and effector CD4 memory T cells were significantly differentially expressed in both subgroups, with higher levels in the high-risk group (**Figure 5A**; **Supplementary Table 7**). Furthermore, the network heat map also showed that the NETosisScore was positively correlated with several CD4 T cells (**Figure 5B**). These data raise the possibility that CD4 T cells were closely associated with the high-risk group in the NETosisScore model and may play an important role in the development of ANCA-GN. Further analysis of CD4 T cell subsets demonstrated that Treg cells exhibited the most substantial correlation and the highest correlation coefficient within the high-risk NETosisScore group (**Figure 5C**). Although Th17, Th2, Th1 were also observed, the strongest association with the high-risk group was seen in Treg. In conclusion, the findings suggest that Treg cells may play a crucial role in the high risk NETosisScore group in ANCA-GN. This supports the notion that Treg cells, which are known to recruit macrophages to the glomerulus, may have a significant impact on disease progression.

Further, to clarify the potential biological functions between the high- and low-risk groups, GSEA enrichment analysis was used to explore the biological processes between the different subgroups. In the high-risk group was mainly in cellular response to biotic stimulus, interleukin-6 production, leukocyte migration and phagocytosis (**Figure 5D**; **Supplementary Table 8**), whereas in the low-risk group was mainly in alpha amino acid metabolism, monocarboxylic acid catabolism and organic acid catabolism (**Figure 5E**; **Supplementary Table 8**), which indicated that the high-risk group might be related to immunity, whereas the low-risk group might be associated with substance metabolism. To validate this conjecture, GSVA analysis was used to investigate the biological pathways between the different subgroups. The heat map of KEGG enrichment analysis showed that the B cell receptor signaling pathways, cytokine-cytokine receptor interaction, Toll-like receptor signaling pathways and NOD-like receptor signaling pathways were enriched in the high-risk group, while other pathways, such as lysine degradation, histidine metabolism and fatty acid metabolism, were more concentrated in the low-risk group (**Figure 5F**; **Supplementary Table 9**). It should be noted that the low-risk group was highly enriched in metabolism-related pathways, whereas the high-risk group was mainly enriched in immune-related pathways, which was consistent with the GSEA enrichment analysis and confirmed the speculation mentioned above. The above results demonstrated that the NETosisScore model could better distinguish the heterogeneity of the immune microenvironment of ANCA-GN, and the high-risk group may contribute to the development of ANCA-GN through immune-related pathways.

### Identification of NETosis-related genes

**Figure 6:**
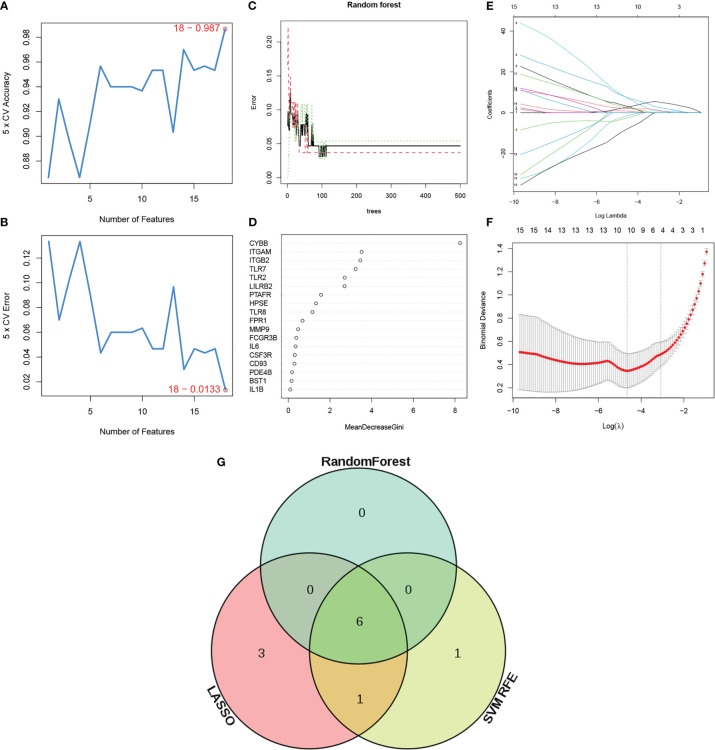
Identification of NETs related genes (NRGs). **(A, B)** Biomarker signature gene expression validation by support vector machine recursive feature elimination (SVM–RFE) algorithm selection. **(C)** Random Forest error rate versus the number of classification trees. **(D)** The top 20 relatively important genes. **(E, F)** Adjustment of feature selection in the minimum absolute shrinkage and selection operator model (lasso). **(G)** Venn diagram of three machine learning algorithms. NRGs: Neutrophil extracellular traps related genes.

To identify NETosis-related genes (NRGs) in ANCA-GN, a series of bioinformatics algorithms were used. To exclude unimportant genes, three machine learning methods were used to select the important genes in ANCA-GN. First, the SVM-RFE algorithm was used to screened out eight genes based on 18 DE-NETs (**Figures 6A, B** **)**. Next, the random forest tree algorithm was applied to identify seven genes (**Figures 6C, D**), while Lasso regression identified 10 NRGs (**Figures 6E, F**). Subsequently, the results of the three machine learning algorithms were crossed, and the final six significant genes were identified as NRGs (CYBB, ITGB2, ITGAM, TLR2, TLR7 and LILRB2) for potential biomarkers of ANCA-GN (**Figure 6G**).

### Analysis of immune characteristics and interaction function of NRGs

**Figure 7:**
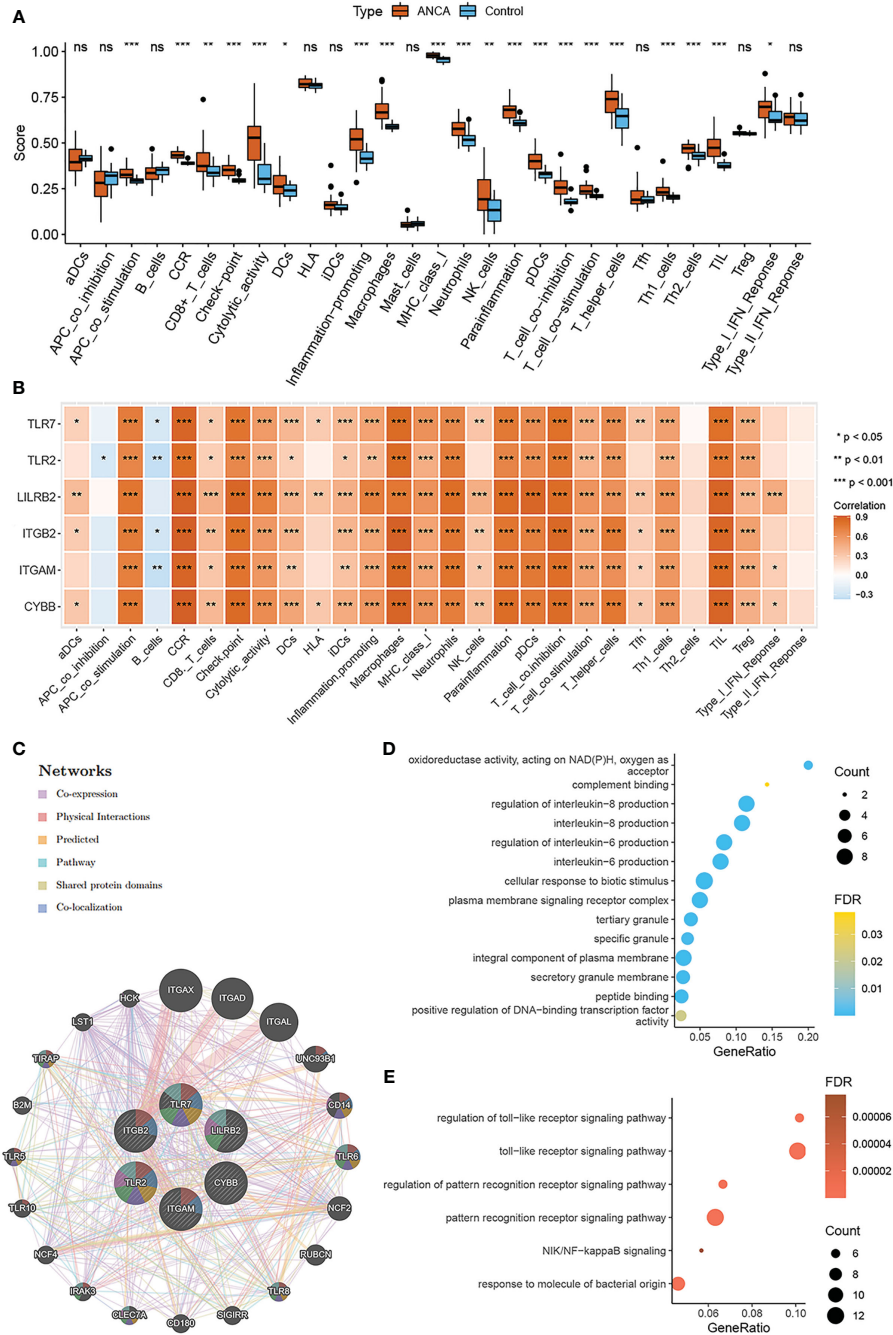
Immune characteristics and interaction function of NRGs **(A)** Comparison of ssGSEA scores of immune cell invasion or immune response between ANCA-GN and healthy controls. **(B)** Correlation between NRGs and immune cell invasion or immune response. **(C)** Characterized gene co–expression network. **(D)** GO analysis of co–expressed genes. **(E)** Co–expressed gene KEGG analysis. *p < 0.05; **p < 0.01; ***p < 0.001; ns, no significant difference.

In order to further explore the immune characteristics of ANCA-GN, the ssGSEA algorithm was employed to predict the differences of immune cell infiltration and immune response between the ANCA-GN group and the control group. mast cells were excluded due to lack of statistical significance in ANCA-GN group and control group, therefore, a total of 28 kinds of immune cell infiltration or immune response were used to explore the differences in immune characteristics between ANCA-GN group and control group (**Figure 7A**). The results revealed that ANCA-GN group exhibited significantly higher levels of CCR, macrophages, T-cell inhibition, and TIL than that of healthy control group (**Figure 7B**). These findings suggested that these immune cells and immune responses play a crucial role in the progression of ANCA-GN. Further, the relationship between six NRGs and 28 immune cells or immune response were further explored. The results uncovered a strong positive correlation between CCR and CYBB (r=0.922; p < 0.001), ITGB2 (r=0.917; p < 0.001), ITGAM (r=0.887; p < 0.001), TLR2 (r=0.836; p < 0.001), TLR7 (r=0.883; p < 0.001), and LILRB2 (r=0.906; p<0.001). Macrophages similarly showed positive correlations with CYBB (r=0.886; p < 0.001), ITGB2 (r = 0.913; p < 0.001), ITGAM (r = 0.862; p < 0.001), TLR2 (r = 0.852; p< 0.001), and TLR7 (r = 0.851; p<0.001). T-cell-co-inhibition also exhibited positive correlations with CYBB (r=0.841; p < 0.001), ITGB2 (r = 0.866; p < 0.001), ITGAM (r = 0.824; p < 0.001), TLR7 (r = 0.822; p < 0.001), and LILRB2 (r = 0.836; p<0.001). TIL was similarly found to be positively correlated with CYBB (r=0.889; p < 0.001), ITGB2 (r = 0.878; p< 0.001), ITGAM (r = 0.848; p < 0.001), TLR7 (r = 0.823; p < 0.001), and LILRB2 (r = 0.888; p<0.001). while APC-co-inhibition was negatively correlated with TLR2 (r=-0.264; p=0.034), and B cells were also negatively correlated with ITGB2 (r = -0.286, p = 0.021), ITGAM (r = -0.360, p = 0.003), TLR2 (r = -0.367, p = 0.002), and TLR7 (r = -0.259, p = 0.038). Our findings reveal a strong association between NRGs and macrophages, while previous research indicates that macrophages, monocytes, and neutrophils are all involved in the NETosis process (16, 27). Macrophages and monocytes contribute to inflammation and tissue damage (28), whereas neutrophils are central to NETs formation, promoting inflammation, endothelial damage, and thrombosis (29). To further elucidate the relationship between NRGs and monocytes, macrophages, and neutrophils in ANCA-GN samples, we employed four algorithms to assess immune cell infiltration abundance: CIBERSORT, xCell, MCPCounter, and quanTIseq. NETosis-related genes primarily include CYBB, ITGAM, ITGB2, TLR2, TLR7, and LILRB2. Subsequently, we visualized the relationship between immune cell infiltration and NRGs using heatmaps. A significant correlation between macrophages and NRGs was observed across all four immune cell infiltration algorithms, with the highest correlation coefficient. Monocytes exhibited a positive correlation with NRGs in CIBERSORT (**Supplementary Figure 2A**) and xCell algorithms (**Supplementary Figure 2B**) but lacked a significant correlation in MCPCounter (**Supplementary Figure 2C**) and quanTIseq algorithms (**Supplementary Figure 2D**). Neutrophils displayed negligible correlation with NRGs. In conclusion, our findings reveal that macrophages display a stronger association with NRGs in ANCA-GN compared to monocytes and neutrophils. These results suggest that the 6 NRGs may act through macrophages in releasing NETs during the progression of ANCA-GN. Further research is needed to fully understand the intricacies of these relationships and their implications for the treatment of ANCA-GN.

when, we delved into the correlation and potential functions of NRGs. The correlation heat map revealed a robust positive correlation between the six NRGs, with values greater than 0.7, signifying a marked functional similarity among the genes (**Supplementary Figure 3**). To further comprehend the relationships among the NRGs, GeneMANIA database were employed to construct a protein-protein interaction network (**Figure 7C**). An analysis of 26 related genes through GO/KEGG enrichment. The biological processes (BP) enriched included regulation of interleukin-8 production and regulation of interleukin-6 production, as well as a cellular response to biotic stimulus. The most enriched cellular components (CC) were secretory granule membrane, integral component of plasma membrane, and tertiary granule. The molecular function (MF) category was enriched with peptide binding, positive regulation of DNA-binding transcription factor activity, and oxidoreductase activity acting on NAD(P)H with oxygen as an acceptor (**Figure 7D**). KEGG analysis showed a main enrichment in the toll-like receptor signaling pathway, the pattern recognition receptor signaling pathway, and the NIK/NF-kappaB signaling (**Figure 7E**). By comparing the functional enrichment pathways of DE-NETs and NRGs, we found that the toll-like receptor signaling pathway was significantly enriched in both.

In an effort to gain deep insights into the molecular mechanism of ANCA-GN, we focused our investigations on the Toll-like receptor signaling pathway. Using the KEGG path view, we uncovered the central role of TLR2 in this process. As shown in (**Supplementary Figure 4B**), through its interaction with peptidoglycan (G+) lipoprotein and zymoglycan, TLR2 binds to downstream NF-kappaB via the P13K-AKt signaling pathway, promoting the synthesis of pro-inflammatory factors such as IL-1B and IL-6, thus exacerbating the progression of ANCA-GN. Furthermore, TLR2 was also found to play a key role in the formation of neutrophil extracellular traps. OxLDL, Staphylococcus aureus, and RSVF protein bind to TLR2 under the influence of HMGB1, activating the Toll-like receptor signaling pathway, which leads to histone acetylation of downstream, finally leading to histone decompression and transcriptional activation. This ultimately leads to NETosis and the capture and elimination of pathogens, as well as thrombosis and coagulopathy, autoimmunity, and complement activation (**Supplementary Figure 4A**). These findings underscore the potential central role of TLR2 in ANCA-GN and its feasibility as an intervention target. Previous research has indicated that the expression of TLR2 and TLR4 is dysregulated in the kidneys of patients with AAV (30), aligning with mounting evidence suggesting a close relationship between Toll-like receptors (TLRs) and the immune response in AAV (31, 32). In the glomeruli of AAV patients, TLR2 and TLR4 expression is significantly elevated compared to healthy controls. Moreover, TLR4 is found to be expressed on glomerular endothelial cells and is negatively correlated with initial serum creatinine (30), the proportion of total crescents, and cellular crescents in kidney specimens, highlighting the crucial role of TLR4 in ANCA-GN.

Our study discovered that TLR4 is upregulated in ANCA-GN patients, with a significantly different expression compared to the normal control group (**Supplementary Figure 5A**). The area under the ROC curve for TLR4 is 0.748, demonstrating considerable diagnostic potential (**Supplementary Figure 5B**). The main affected pathways include downregulation of bile acid metabolism and upregulation of mitotic spindle, IL6-JAK-STAT3 signaling, inflammatory response, and PI3K-AKT-mTOR signaling (**Supplementary Figure 5C**). Immune cell infiltration analysis revealed that TLR-4 is significantly positively correlated with macrophages and Th1 cells and negatively correlated with immature dendritic cells (**Supplementary Figure 5D**). Additionally, TLR4 exhibits a significant positive correlation with serum creatinine (Scr) (**Supplementary Figure 5E**) and a significant negative correlation with estimated glomerular filtration rate (eGFR) (**Supplementary Figure 5F**). In conclusion, TLR4 appears to play a critical role in ANCA-GN, as it is upregulated in the condition and exhibits significant diagnostic power. The molecule is involved in several key pathways and correlates with specific immune cell infiltration, as well as renal function. These findings suggest that TLR4 may be an important therapeutic target and prognostic biomarker for ANCA-GN.

### Construction of nomogram and analysis of clinical renal function

**Figure 8:**
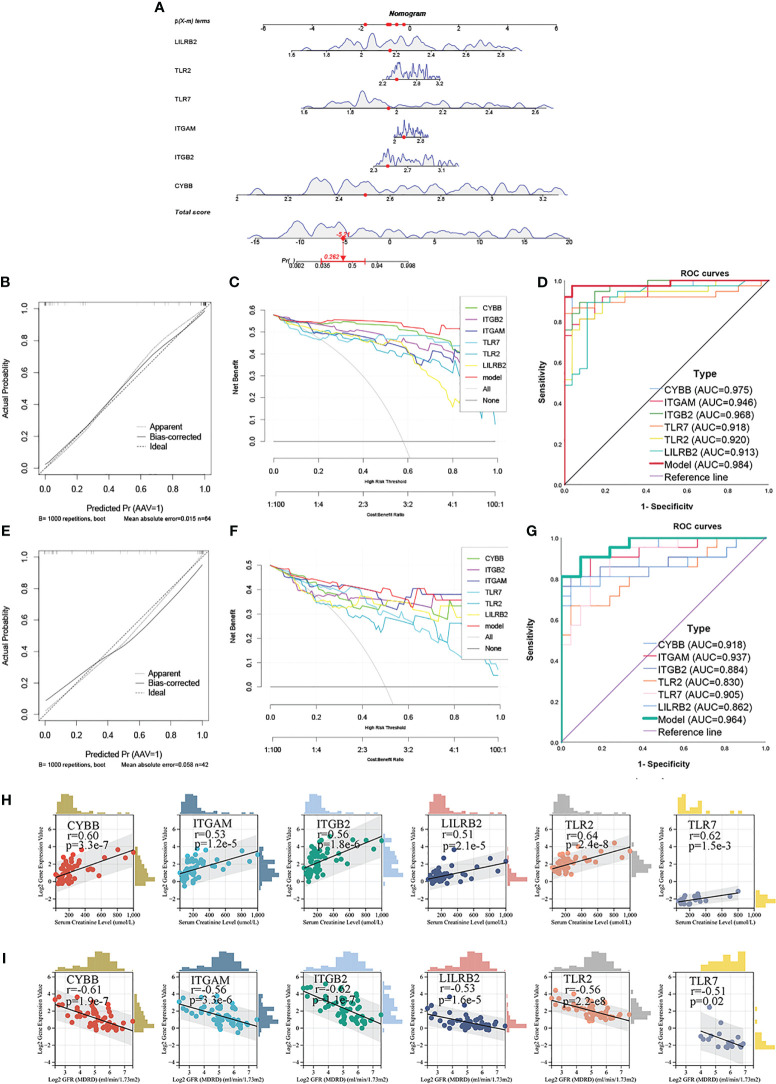
Construction of nomogram and analysis of clinical renal function **(A)** Nomogram to predict the occurrence of ANCA-GN. **(B)** Calibration curves were used to evaluate the calibration of the model. **(C)** The clinical decision benefit of the model was assessed using the DCA curve. **(D)** ROC curve was used to evaluate the clinical discrimination of the model. **(E–G)** Calibration curve, DCA curve and ROC curve of the evaluation model in the GSE108112 set. **(H)** Scatter diagram of the correlation between 6 NRGs and glomerular filtration rate. **(I)** Scatter diagram of the correlation between 6 NRGs and serum creatinine level.

In order to explore the role of NRGs in clinical practice, the clinical diagnostic efficacy of NRGs and its relationship with renal function were separately explored. First, a clinical diagnostic model for ANCA-GN was constructed based on the expression levels of 6 NRGs (CYBB, ITGB2, ITGAM, TLR2, TLR7, and LILRB2) in the training sets (GSE104948 and GSE108109) using the “rms” package in R language (**Figure 8A**). Then, the diagnostic efficacy of the model was evaluated through calibration curve analysis, decision curve analysis (DCA), and receiver operating characteristic (ROC) curve analysis. In the training set, the calibration curve showed a minimum difference between the actual risk of ANCA-GN and the predicted risk, which highlight the practicality of the model in predicting ANCA-GN (**Figure 8B**). The DCA showed that when the high-risk threshold of the DCA curve was 0-1, the model was significantly higher than other single NRGs, indicating that the model was significantly higher in predicting the decision benefits of ANCA-GN than other single NRGs, and that patients could benefit from the model (**Figure 8C**). Additionally, the ROC curve showed that the area under the curve (AUC) of the model was approximately 0.984, higher than the AUC of any other single gene, indicating a high diagnostic efficiency of the model (**Figure 8D**). The independent dataset (GSE108112) was used to validate the diagnostic efficacy of the model. Clearly, the results of the calibration curve, DCA curve analysis, and AUC of the ROC curve of the model were consistent with the training dataset (**Figures 8E–G**). Thus, the clinical diagnostic model based on NRGs has strong diagnostic efficacy and can provide an effective reference for predicting the occurrence of ANCA-GN.

Subsequently, the correlation between NRGs and clinical renal function was further analyzed. Expression levels of 6 NRGs, corrected glomerular filtration rate (GFR) and serum creatinine (Scr) were downloaded from the Nephroseq database V5. Spearman correlation analysis revealed that the expression levels of the 6 NRGs increased as renal function declined, and all NRGs were positively correlated with GFR. The strongest correlation was found between TLR2 and GFR (r = 0.64, p = 2.4e-8) (**Figure 8H**). Additionally, all 6 NRGs were negatively correlated with Scr levels (**Figure 8I**), suggesting that NRGs can predict the severity of renal injury of ANCA-GN and may be involved in the progression of renal damage of ANCA-GN. These findings suggest that NRGs may serve as potential biomarkers for the prognosis of ANCA-GN.

### Independent dataset verification of NRGs

**Figure 9:**
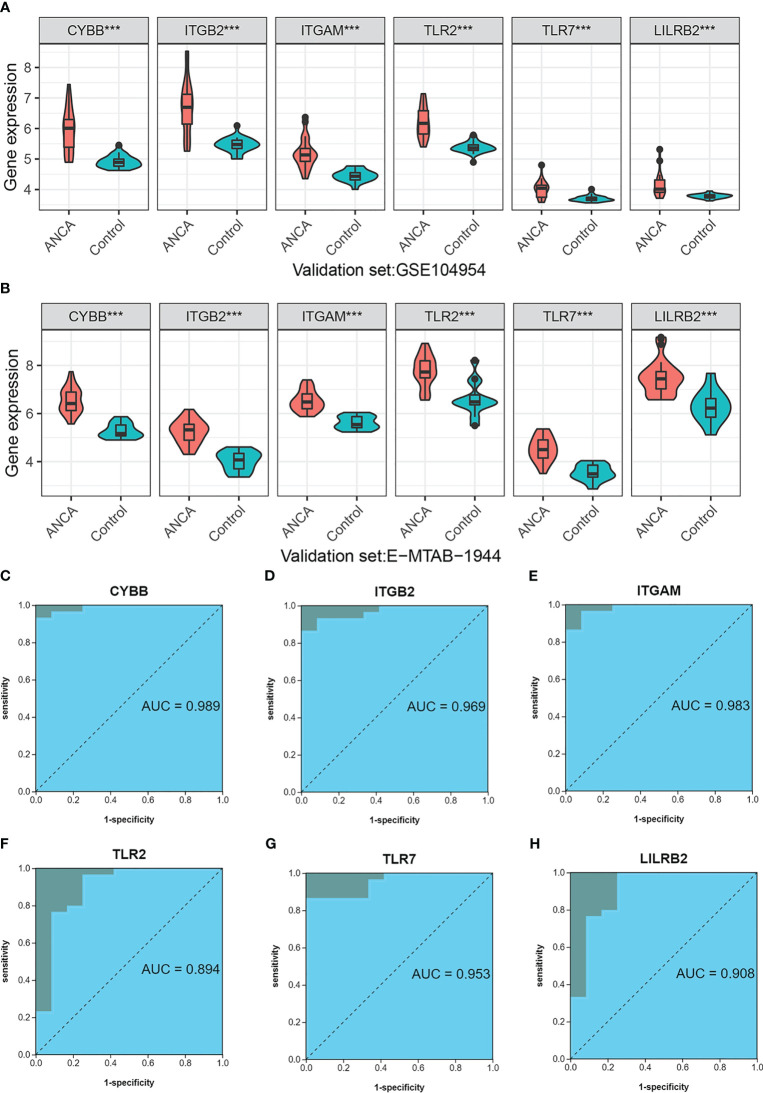
Independent dataset Verification of NRGs **(A)** Differential expression of NRGs in an independent dataset GSE104954. **(B)** Differential expression of NRGs in an independent dataset E-MTAB-1944. **(C–H)** ROC curve of NRGs in independent data set E-MTAB-1944. ***p < 0.001.

When it comes to being biomarkers of diseases, it’s not enough for them to simply have differential expression between healthy and control groups. The markers must also be highly effective in diagnosis. To assess the suitability of the six NRGs in this regard, we performed an analysis. Our findings from the training dataset indicated that the six NRGs showed a statistically significant difference in expression between the ANCA-GN group and the normal control group (p<0.001), with elevated expression levels observed in the ANCA-GN group (**Supplement Figure 6A**). The results from independent validation study further supported these observations, with differential expression of the six NRGs detected in both GSE104954 (p<0.001) (**Figure 9A**) and E-MTAB-1944 (p<0.001) (**Figure 9B**). Additionally, all NRGs were found to exhibit higher expression in the ANCA-GN group compared to the control group. In conclusion, the six NRGs satisfy the criteria for differential expression between ANCA-GN and normal control groups, making them potential biological markers of ANCA-GN.

Next, the diagnostic value of 6 NRGs in ANCA-GN and normal samples was further investigated. In the training dataset, all NRGs showed a high level of diagnostic accuracy as indicated by the areas under the receiver operating characteristic (ROC) curve (AUC), which were all greater than 0.90 (**Supplementary Figure 6B**). These NRGs include CYBB (AUC: 0.975), ITGB2 (AUC: 0.968), ITGAM (AUC: 0.946), TLR2 (AUC: 0.920), TLR7 (AUC: 0.918), and LILRB2 (AUC: 0.913). This trend was confirmed in the independent validation set E-MTAB-1944, where CYBB demonstrated an AUC of 0.989, followed by ITGB2 (AUC: 0.969), ITGAM (AUC: 0.983), TLR2 (AUC: 0.83), TLR7 (AUC: 0.953), and LILRB2 (AUC: 0.908) (**Figures 9C-H**). These findings were further supported by the results from the independent dataset GSE104954 (**Supplementary Figure 6C**). These results suggest that NRGs have a strong potential as biological markers for ANCA-GN, as they exhibit excellent diagnostic performance in distinguishing between ANCA-GN and healthy samples.

### qPCR and immunohistochemistry staining

**Figure 10:**
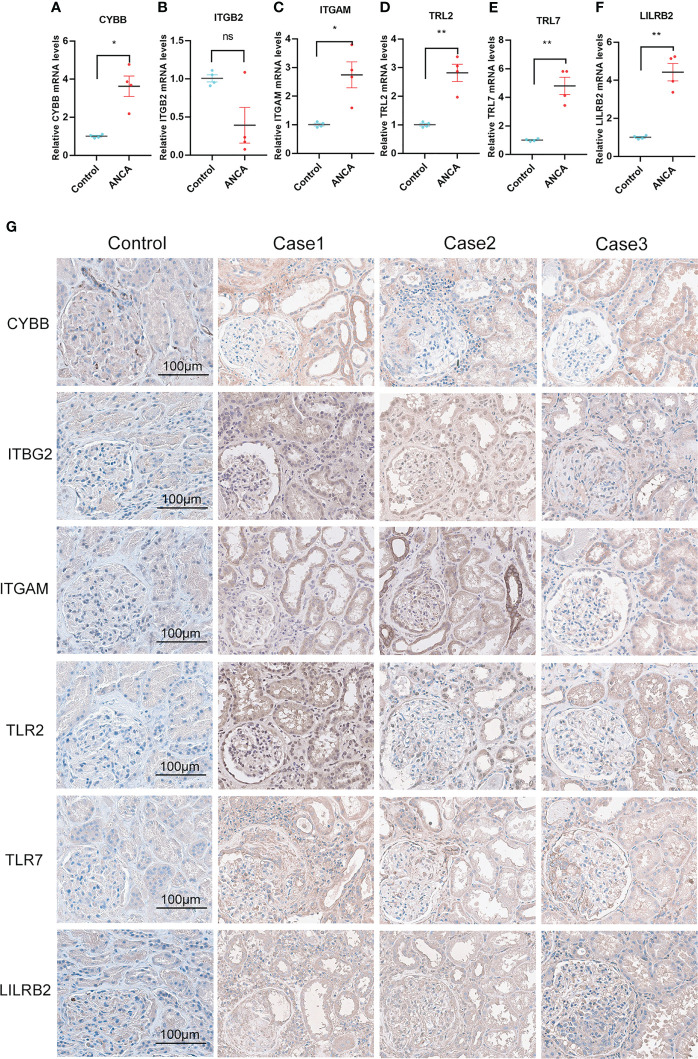
qPCR and Immunohistochemical Staining Validation of NRGs. **(A-F)** qPCR analysis of NRGs in whole blood samples from ANCA-GN patients and healthy individuals. **(G)** Immunohistochemical staining of NRGs in renal tissues from ANCA-GN patients and adjacent renal tissues in patients with renal clear cell carcinoma, Magnification (200x), scale bar 100 μm. *p < 0.05; **p < 0.01; ns, no significant difference.

To translate the potential value of NRGs into a clinical setting, we determined the differential expression levels of 6 NRGs between ANCA-GN patients and healthy controls. Whole blood samples were collected from clinically diagnosed ANCA-GN patients and healthy individuals that were matched for age and gender, and the expression levels of 6 NRGs were quantified using qPCR. The qPCR results indicated that with the exception of ITGB2, all NRGs were significantly overexpressed in ANCA-GN patients compared to healthy controls, and the expression levels were found to be statistically significant (p<0.01) between the ANCA-GN group and the healthy group (**Figures 10A-F**). This human-level validation of the mRNA expression of 6 NRGs was consistent with the results of both the training dataset and the independent validation set, suggesting that NRGs could serve as potential markers of ANCA-GN progression.

Subsequently, we attempted to perform immunohistochemistry (IHC) staining for NRGs (CYBB, ITGB2, ITGAM, LILRB2, TLR2, and TLR7) in kidney sections from ANCA-GN patients and age- and sex-matched ccRCC patients with adjacent non-tumor tissues. The results of IHC staining revealed that CYBB displayed moderate positivity (score = 2+) in ANCA-GN, mainly in the glomerular endothelial cells and tubular epithelial cells. Interestingly, although ITGB2 did not show significant differential expression in qPCR, it exhibited moderate positivity (score = 2+) in ANCA-GN. This discrepancy might be due to the small sample size, which may not fully reflect the actual differences and requires validation with more samples. In contrast, IHC staining allows for the observation of protein localization within cells, possibly enriching on the surface of glomerular and tubular endothelial cells in inflamed areas, resulting in moderate positivity. ITGAM showed strong positivity in ANCA-GN, primarily in the glomerular and tubular endothelial cells. TLR2 and TLR7 staining exhibited strong positivity (score = 3+) in ANCA-GN, mainly in the tubular epithelial cells and glomerular endothelial cells. LILRB2 staining displayed moderate positivity (score = 2+) in ANCA-GN, predominantly in glomerular and tubular cells (**Figure 10G**). This experiment confirms that, consistent with high-throughput sequencing data and qPCR results, NRGs are significantly overexpressed in ANCA-GN and may play an essential role in ANCA-GN.

## Discussion

ANCA-associated glomerulonephritis (ANCA-GN) is an autoimmune disease, the etiology and pathogenesis of which remain incompletely understood. Increasing evidence suggests that neutrophil extracellular traps (NETs) play an important role in the pathogenesis and pathological processes of ANCA-GN. As a neutrophilic immune defense mechanism, NETs can effectively remove pathogens and promote wound healing to a certain extent. However, when NETs are excessively activated and released, the aggregation of extracellular DNA and the release of inflammatory factors such as tumor necrosis factor-α (TNF-α), interleukin-1β (IL-1β), leading to inflammatory responses and cell damage (33). Some studies have shown that ANCA can promote NETosis and release self-antigens, inducing autoimmune responses and leading to inflammation and damage of the glomerulus in ANCA-GN (34). Therefore, exploring the role of NETosis in ANCA-GN is of great significance for further research into the pathogenesis and treatment of ANCA-GN. In present study, we explored the potential mechanisms of ANCA-GN patients with different risks and identified prognostic biomarkers associated with prognosis based on DE-NETs (Differentially expressed NETs genes). Firstly, 18 DE-NETs were identified by WGCNA, and then the NETosisScore model was created using the ssGSEA algorithm. The findings indicate a positive correlation between the NETosisScore and the prevalence of high-risk patients. Notably, the proposed model effectively discriminated ANCA-GN patients from healthy controls, with individuals in the high-risk category exhibiting an elevated immune pathway enrichment and those in the low-risk category showing a higher metabolic pathway enrichment. Moreover, individuals classified as high-risk demonstrated a robust immune response and pronounced immune cell infiltration. Finally, six significantly related NRGs (NETosis-related genes) were identified by three machine learning algorithms based on 18 DE-NETs. The diagnostic model constructed from NRGs had a significant predictive ability. NRGs levels increased with the decline of kidney function and were correlated with Scr and GFR, making them potential prognostic biomarkers. Quantitative PCR analyses revealed significant variations in the expression levels of numerous NRGs in patients with ANCA-GN.

Through weighted gene co-expression network analysis and variation analysis, 18 differentially expressed neutrophil extracellular traps (DE-NETs) were identified in ANCA-GN. To elucidate the potential biological functions of these DE-NETs in ANCA-GN, GO enrichment analysis was performed, and the result revealed that they were primarily associated with the positive regulation of cytokines such as IL6 and IL8. In a randomized controlled study of 78 AAV patients over 18 months, the concentration of sIL-6 was found to be correlated with baseline PR3-AAV titers, and an increase in sIL-6 concentration during remission was subsequently associated with severe relapse in patients receiving rituximab (RTX) therapy (35). In another study involving 186 active AAV patients treated with RTX, eight cytokines, including IL6 and IL8, were found to be significantly higher in PR3-AAV than in MPO-AAV after RTX treatment, indicating that cytokines such as IL6 and IL8 may play an important role in ANCA-GN (36). In addition to neutrophil extracellular trap formation, KEGG pathway enrichment analysis revealed that the toll-like receptor signaling pathway and Staphylococcus aureus infection pathway were mainly enriched in DE-NETs. O’Sullivan et al. found that the expression of TLR4 and TLR2 in the kidneys of AAV patients was negatively correlated with glomerular filtration rate (37). Furthermore, some studies have shown that plasmid-encoded peptides from Staphylococcus aureus can induce autoimmune glomerulonephritis by inducing anti-myeloperoxidase antibodies (38), indicating that toll-like receptor signaling pathway and Staphylococcus aureus infection may be involved in the pathogenesis and development of ANCA-GN.

In an effort to quantify the extent of NETosis in each ANCA-GN patient, the ssGSEA algorithm was used to construct the NETosisScore based on 18 differentially expressed NETs. Then ANCA-GN patients were categorize into high-risk group and low-risk group based on the NETosisScore, and the differences in gene expression, immune cell infiltration, and biological function between the two groups were explored. The results of our study demonstrate that the NETosisScore model exhibited significant distinctions not only between patients diagnosed with ANCA-GN and those in the control group but also displayed a progressive escalation in the high-risk cohort as the NETosisScore increased. Moreover, all DE-NETs displayed significant differences in expression between the high-risk group and low-risk group, and further validation through ROC curves and PCA analysis suggested that the model has a strong ability to differentiate between the two groups. As regard to immune cell infiltration, our results showed that the high-risk group exhibited characteristics of high immune activation, similar to the high immune infiltration observed in cases with poor prognosis previously (39). This prompted us to speculate that there is heterogeneity in immune infiltration in ANCA-GN, with the high-risk group potentially consisting of immune individuals and the low-risk group consisting of non-immune individuals. Interestingly, the biological functions predicted by the GSVA algorithm between the high-risk group and low-risk group were different. The KEGG results disclosed that the high-risk group was significantly enriched in B cell receptor signaling pathways, cytokine receptor interaction, toll-like receptor signaling pathways, and nod-like receptor signaling pathways, while other pathways such as lysine degradation, histidine metabolism, and fatty acid metabolism are more concentrated in the low-risk group. Notably, the low-risk group exhibits greater enrichment in metabolism-related pathways, while the high-risk group exhibits greater enrichment in immune-related pathways. GSEA enrichment analysis yielded similar results. All these findings supported our hypothesis that NETosisScore grouping can better differentiate the heterogeneity of the immune microenvironment in ANCA-GN, with high-risk and low-risk groups possibly shaped by different mechanisms.

In this study, we conducted classification of ANCA-GN based on the median value of NETosisScore. Immunocellular infiltration analysis revealed that patients in the high NETosisScore group exhibited pronounced immunocellular infiltration and heightened immune response, thereby providing compelling evidence for the efficacy of NETosis in the classification of ANCA-GN. However, during the correlation analysis of CD4T cells at different risk levels based on NETosisScore, we observed a significant positive correlation between the high-risk group of NETosisScore and Treg cells. This result contradicts previous research, which has suggested that T regulatory cells in the context of AAV are associated with reduced inflammation. Treg cells play a crucial role in preventing lethal autoimmune inflammation, and their immunosuppressive function relies on the sustained expression of the transcription factor Foxp3. A study revealed that Foxp3-positive T cells in the renal interstitium may serve as a predictor of kidney survival in patients with MPO-AAV (40). Studies have revealed that when Treg function is intact, Treg cells have the ability to continuously reset immune homeostasis (41). However, there is compelling evidence indicating the impairment of Treg cell function in AAV (42, 43). In this study, we observed a significant correlation between Treg cells and the high-risk group of NETosisScore. We speculate that the pronounced immune cell infiltration observed in patients from the high-risk group of NETosisScore may be associated with Treg cell dysfunction. In this scenario, impaired Treg cells fail to exert their normal regulatory function in MPO-AAV, potentially leading to an ineffective suppression of the inflammatory response and exacerbation of tissue damage. Moreover, we also observed a significant correlation between M2 macrophages and the high-risk group of NETosisScore. M2 macrophages play a role in regulating immune responses and promoting immune tolerance and homeostasis (44). In the context of AAV, several *in vitro* and histological studies have demonstrated an increase in M2 macrophages expressing CD206 and CD163 (45). Additionally, research has suggested that Treg cells can recruit M2 macrophages to facilitate tissue repair following damage (46). Another possible explanation for the significant positive correlation between the high-risk group of NETosisScore and Treg cells in this study could be that in ANCA-GN, there is a need for Treg cells to recruit more M2 macrophages to facilitate the healing of tissue damage. In summary, we speculate that the reduction or dysfunction of Treg cells in patients with high-risk NETosisScore in ANCA-GN exacerbates the progression of the disease. Additionally, Treg cells may further recruit M2 macrophages for renal glomerular repair. However, the precise mechanisms underlying these processes require further experimental validation.

Based on 18DE-NETs, six NRGs were identified using three machine learning algorithm, including CYBB, ITGB2, ITGAM, TLR2, TLR7, and LILRB2. The CYBB gene, located on the X chromosome, encodes the gp91phox subunit of the cytochrome b558, which is a transmembrane component of the NADPH oxidase complex. Studies have suggested that CYBB is differentially expressed in AAV, but its specific pathogenesis still was not clear (47). CYBB is an essential constituent of the phagocytic cell membrane-bound oxidase that generates superoxide. Nevertheless, studies investigating the correlation between CYBB and autoimmune disorders such as Inflammatory Bowel Disease are scarce (48–50). there are few studies on ITGB2 and AAV. Genetic variations in the ITGB2 gene encoding the β2 integrin subunit are believed to cause leukocyte adhesion deficiency 1 (51). Toll-like receptors (TLRs), the most characteristic members of pattern recognition receptors (PRRs), play a significant role in innate immunity. Previous research has revealed a marked rise in TLR-2 expression in AAV patients relative to healthy controls (30). Additionally, the association between TLR2 expression in the kidneys of AAV patients and renal injury indicates that TLR2 could represent a promising therapeutic target for the treatment of this disease (37). Recently, circulating high mobility group box 1 (HMGB1) has been found to reflect the disease activity of AAV, and HMGB1 can affect the formation of NETs by interacting with TLR2, TLR4, and RAGE (52). Studies have shown that the TLR2 signaling pathway is involved in the development of Th17-driven immune responses, which is consistent with the observed Th17 cell phenotype in granulomatosis with polyangiitis (32). In Systemic Lupus Erythematosus patients, the loss of self-antigen tolerance in B cells is controlled by toll-like receptors inside the cells, and TLR7 drives follicular outside B cell responses and germinal center reactions, which are associated with the production of autoantibodies and the pathogenesis of the disease (53). In GPA, studies have shown that PR3-ANCA activates human monocytes to produce cytokines through up-regulating TLR and Nod signaling pathways under the stimulation of various microbial components, which may partially participate in the inflammatory process of GPA (54). As a member of the leukocyte immunoglobulin-like receptor (LILR) family, LILRB2 mainly exists on myeloid immune cells by binding to MHC-I and HLA-G (a non-classical class I molecule) and plays an important role in providing negative feedback for inflammatory responses (55). LILB2 has been found to regulate immune responses in the middle and late activation stages of neutrophil life and can inhibit the degranulation and phagocytosis of neutrophils by cross-linking with HLA-G (56). The ITGAM gene encodes integrin CD11b, an α chain of the integrin heterodimer CD11B/CD18, which is highly expressed in leukocytes and regulates TLR-dependent pro-inflammatory signaling. The three non-synonymous Nucleotide diversity of ITGAM, SNPs, SLE and LN, are at increased risk and strongly associated with IFN I levels (57). The relationship between ITGAM and systemic sclerosis has also been reported (58). Therefore, NRGs are closely related to ANCA-GN and may be involved in the development and progression of ANCA-GN.

The biological functions, immunological properties, and clinical value of NRGs in ANCA-GN are further explored. The GO enrichment analysis of NRGs was conducted based on GeneMANIA database, and it was found that the regulation of IL-6 and IL-8 production is consistent with functional enrichment of DE-NETs, indicating that IL-6 and IL-8 may play a key role in ANCA-GN. The KEGG analysis revealed significant enrichment in the toll-like receptor signaling pathway and the NIK/NF-kappaB signaling pathway, which is consistent with prior research. The toll-like receptor signaling pathway plays a critical role in the onset and progression of ANCA-GN (59). By combining KEGG Pathview, it is hypothesized that Staphylococcus aureus binds to TLR2 under the action of HMGB1 and acts on downstream histone acetylation through the toll-like receptor signaling pathway, then promoting histone decondensation and transcriptional activation, ultimately promoting NETosis, leading to pathogen capture and killing, thrombosis and coagulation, autoimmunity, and complement activation. Although this hypothesis allows NF-kappaB, toll-like receptor signaling pathway and other pathways to be orderly connected with NETosis through the TLR2 core regulatory network, it still requires a large amount of clinical and basic experiments to verify. In the analysis of the relationship between NRGs and immune cell infiltration, the results showed that CCR, macrophages, T-cell inhibition, and TIL were not only significantly higher in the ANCA-GN group than in the healthy control group, but also significantly positively correlated with all NRGs, indicating that NRGs may play a role in the progression of ANCA-GN through T cells and macrophages and their related immune reactions. In order to explore the clinical value of NRGs, a clinical diagnosis model of ANCA-GN was constructed, and NRGs were found to have good performance in distinguishing healthy samples from ANCA-GN in an independent dataset, providing effective reference value for clinical decision-making. To further explore the correlation between NRGs and clinical renal function, the expression level and clinical data of 6 kinds of NRGs in Nephroseq database were analyzed, and the result showed that NRGs were positively correlated with glomerular filtration rate and negatively correlated with serum creatinine. These findings suggested that NRGs may predict the severity of ANCA-GN renal injury and may be involved in its progression. Given the significance of NRGs in ANCA-GN, qPCR validation of five NRGs in ANCA-GN patients and normal controls was conducted, highlighting their potential value as diagnostic and prognostic biomarkers for ANCA-GN.

At the same time, this study has several limitations. Firstly, due to the lack of clinical data for each patient, the relationship among the NETosisScore model, NRGs and clinical characteristics of ANCA-GN could not be explored. Secondly, although this study included samples from multiple gene chips, the sample size is still relatively small, and it still needs a lot of research. In addition, the investigation of the role of NRGs in ANCA-GN is mainly conducted through bioinformatics and validated by qPCR, and further basic experimental still await further investigation.

To sum up, our study systematically explored the potential mechanism of AAV through comprehensive bioinformatics analysis. At the same time, this study also screened some key genes and important pathways, which may help to find new biomarkers or therapeutic targets in ANCA-GN. In order to explore the pathophysiological mechanism of ANCA-GN more targeted. In the future, further animal and clinical molecular biology experiments are needed to verify the results of this study.

## Data availability statement

The datasets presented in this study can be found in online repositories. The names of the repository/repositories and accession number(s) can be found in the article or **Supplementary Material**.

## Ethics statement

The studies involving human participants were reviewed and approved by the human experiments involved in this study were examined and approved by the Ethics Committee of Zhongnan Hospital of Wuhan University. Written informed consent for participation was not required for this study in accordance with the national legislation and the institutional requirements.

## Author contributions

MT participated in experimental design, data analysis, and article writing. LL drafted the conception and design of the article. YL performed part of the experimental data collection and analysis. WL, HN and YH participated in the conception of the article. PG designed the study and provided writing guidance. All authors contributed to the article and approved the submitted version.
