# Supplementary material for: Identification and validation of immune-associated NETosis subtypes and biomarkers in anti-neutrophil cytoplasmic antibody associated glomerulonephritis
Source: Front Immunol. 2023 Jul 3;14:1177968. doi: 10.3389/fimmu.2023.1177968 (PMC10351423; doi:10.3389/fimmu.2023.1177968)
Supplement: Supplementary file 1 [file DataSheet_1.docx]

***Supplementary materials***


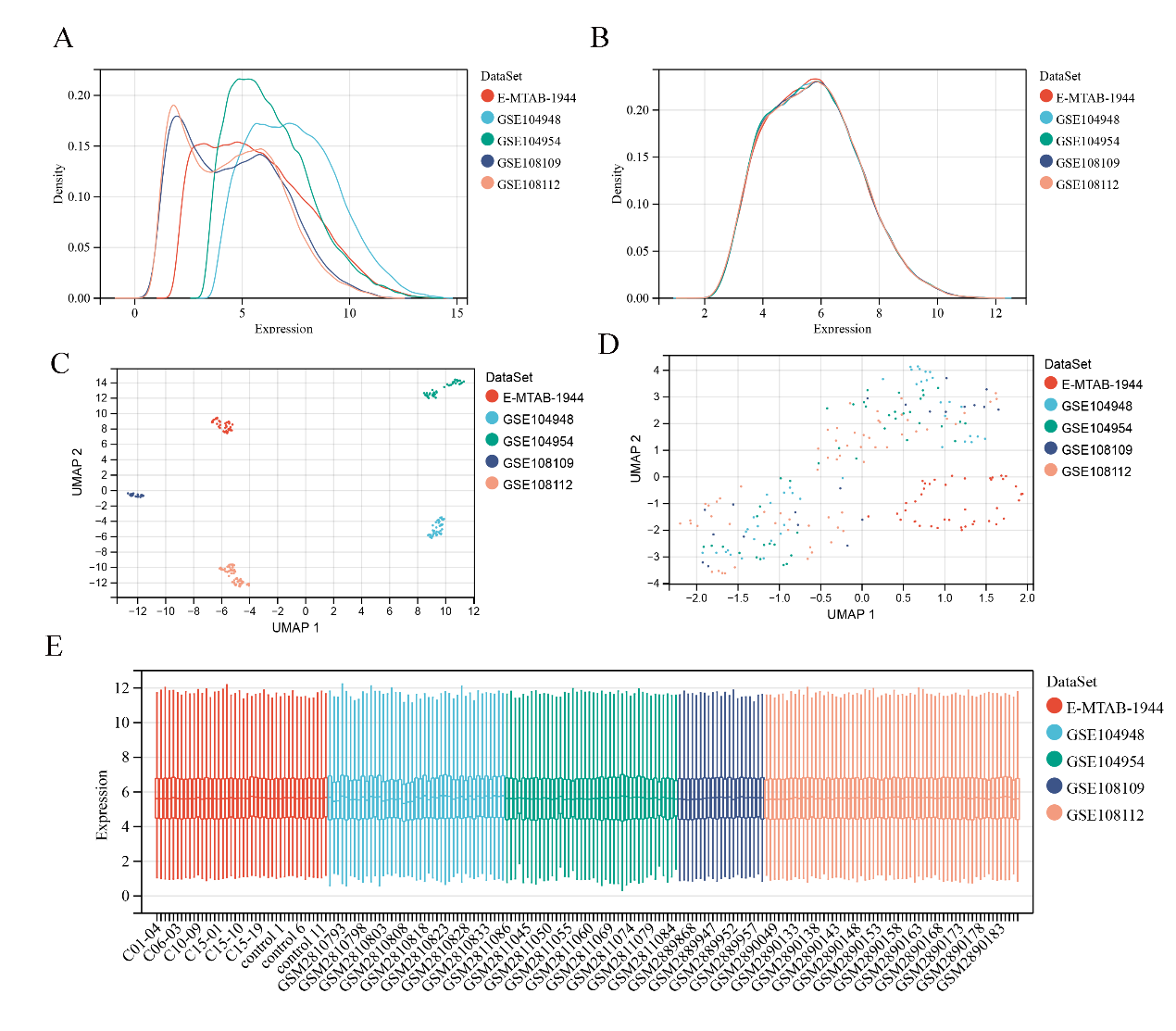


**Supplementary Figure 1 Normalization process.** (A) UMAP plot of the five datasets before normalization. (B) UMAP plot of the five datasets after normalization. (C) Expression density plot of the five datasets before normalization. (D) Expression density plot of the five datasets after normalization. (E) Expression distribution plots for the five datasets after normalization. UMAP, Uniform Manifold Approximation and Projection.


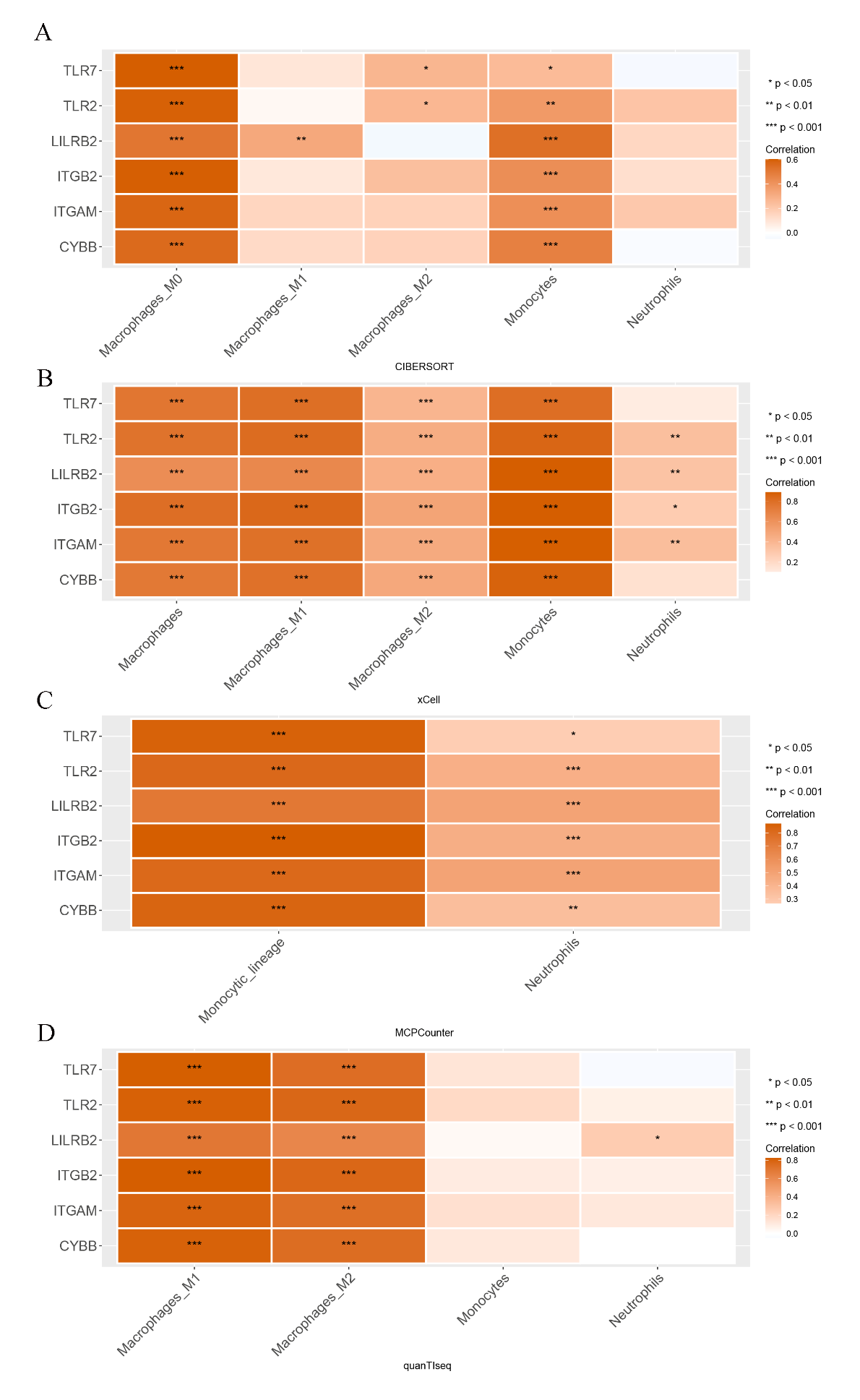
 **Supplementary Figure 2 Analyzing the correlation between macrophages, monocytes, and neutrophils with NRGs based on four immune cell infiltration algorithms.** (A) CIBERSORT. (B) xCell. (C) MCPCounter. (D) quanTIseq.


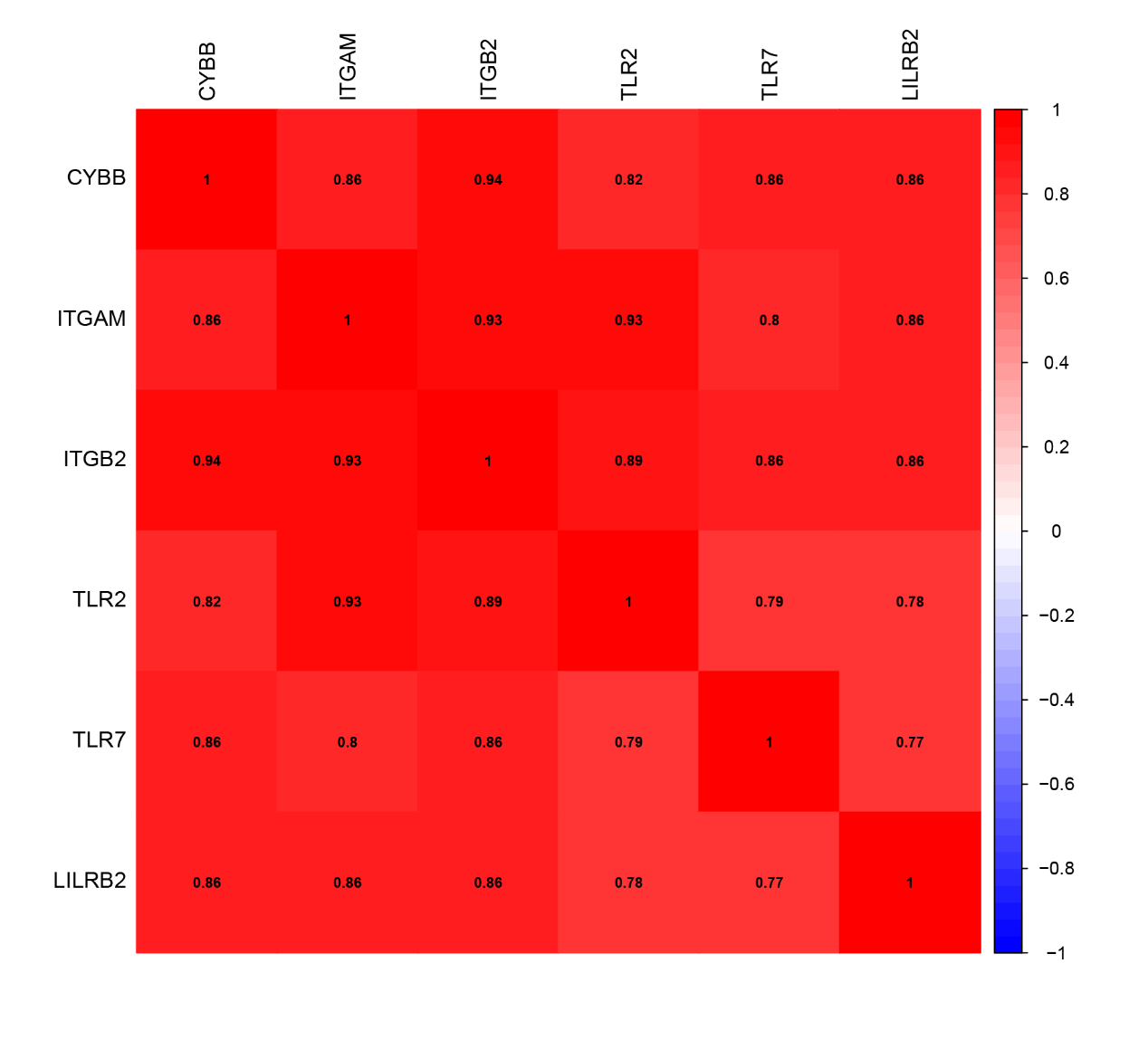
 **Supplementary Figure 3 Correlation between NRGs.** NRGs, NETosis-related genes


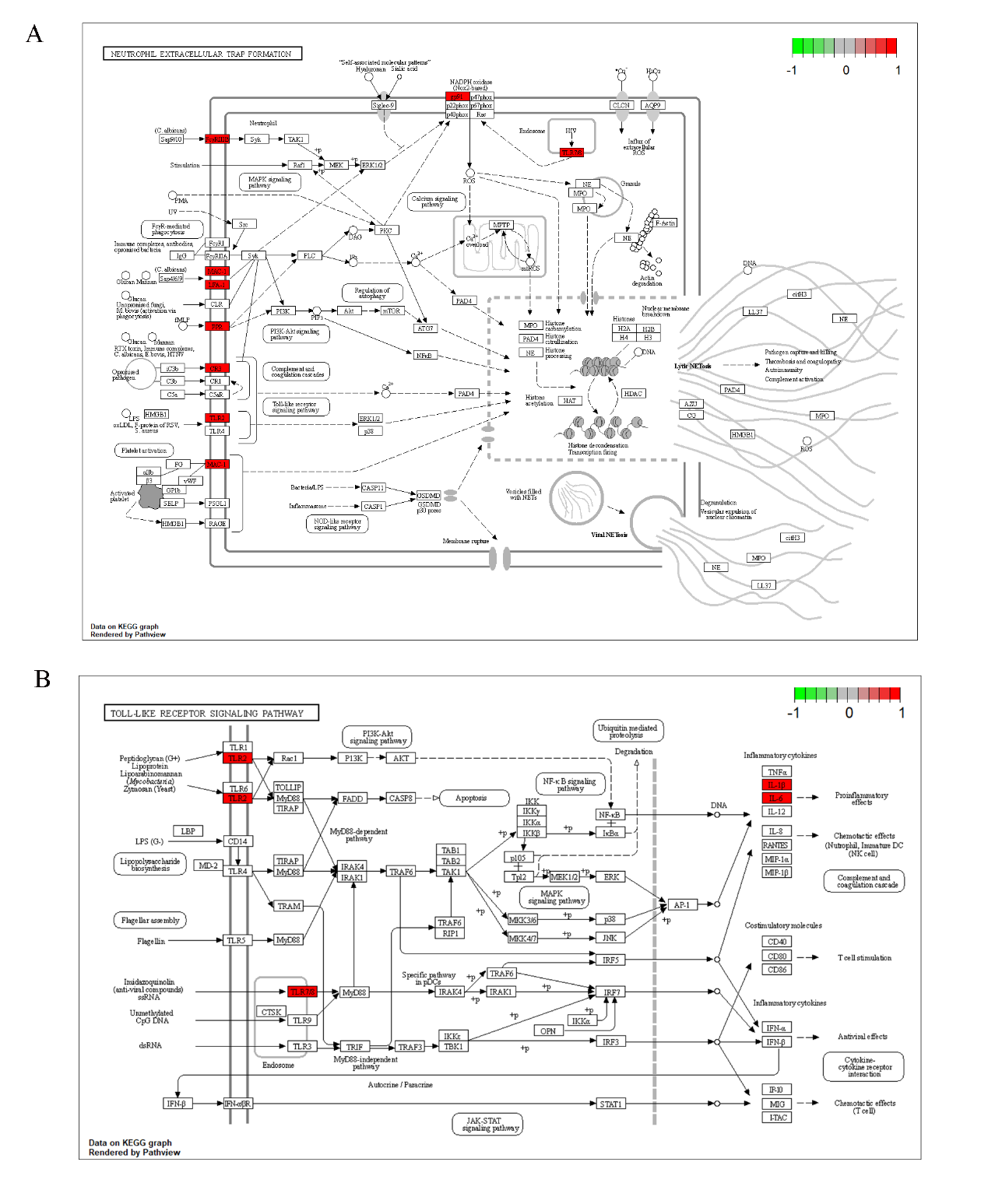


**Supplementary Figure 4 KEGG pathview based on DE-NETs** (A). Neutrophil extracellular trap formation. (B). Toll-like receptor signaling pathway.


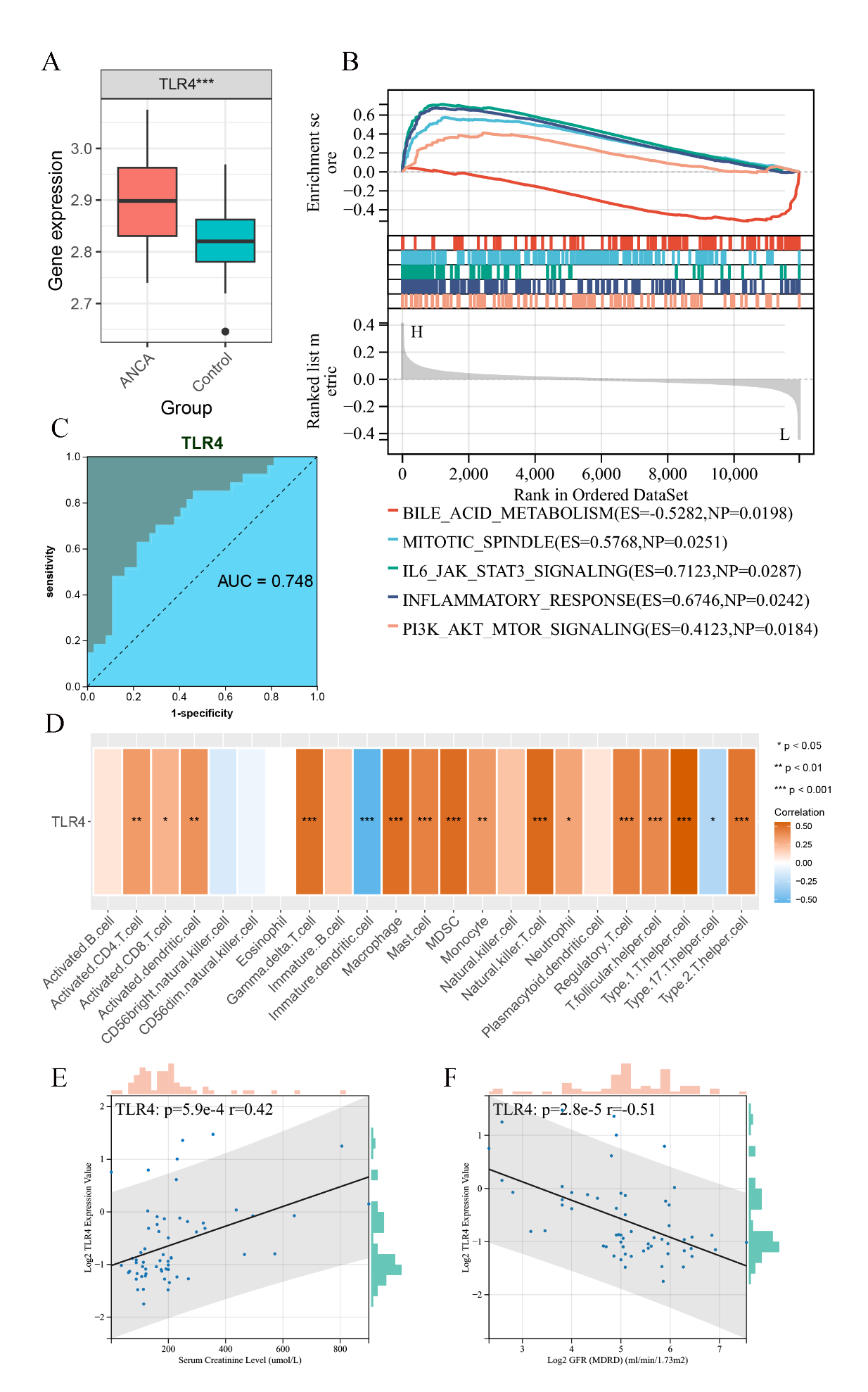
 **Supplementary Figure 5 TLR-4 in ANCA-GN** (A) Box Plot of ANCA-GN Patients and Control Group. (B) Gene set enrichment analysis (GSEA) was performed by stratifying samples into high-expression (≥50%) and low-expression (<50%) groups based on TLR4 expression levels. The h.all.v7.4.symbols.gmt subset was obtained from the Molecular Signatures Database. Statistical significance was determined with a p-value of <0.05 and a false discovery rate (FDR) of <0.25. (C) ROC curve of TLR4 in training set. (D) Correlation analysis between TLR4 and immune cell infiltration using the CIBERSORT algorithm. (E-F) Exploring the Correlation between TLR4, Creatinine, and eGFR Using the Nephroseq v5 Database.


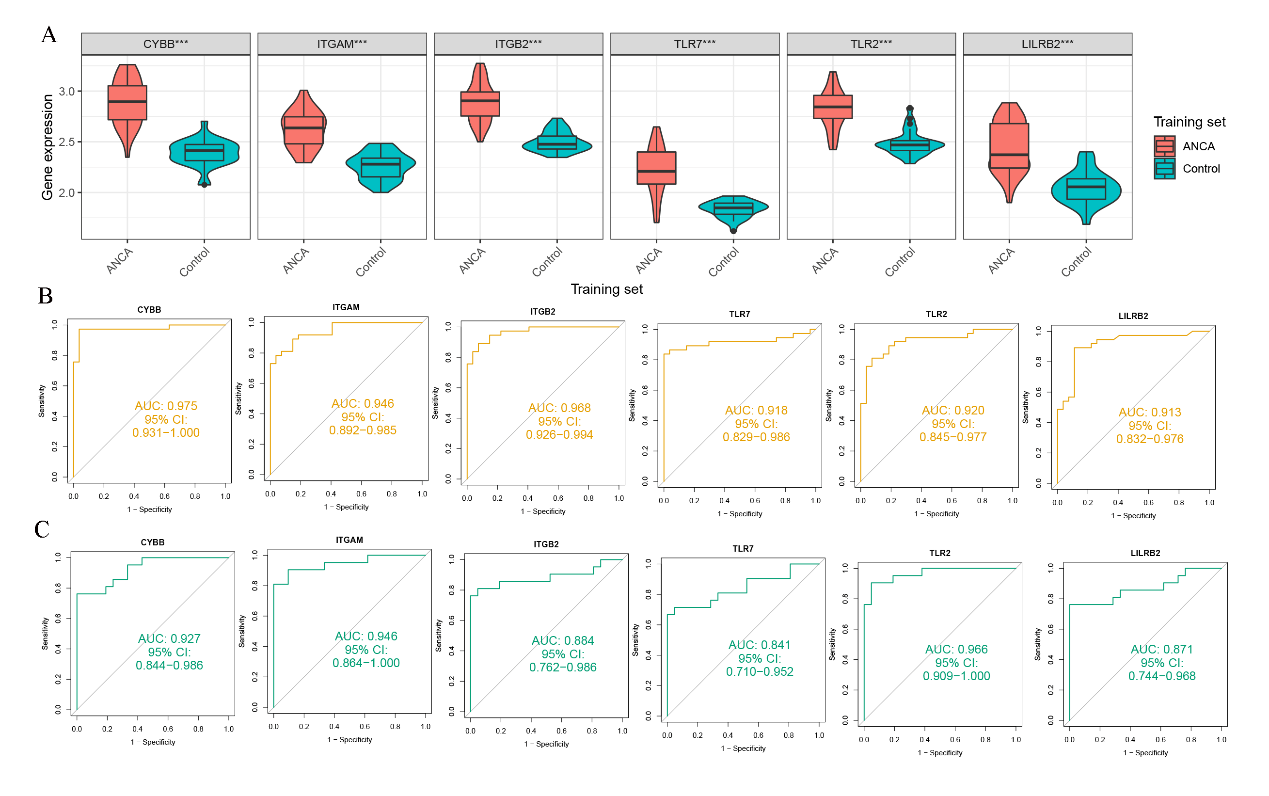


**Supplementary Figure 6 NRGs validation of gene expression and roc curve.** (A) Differential expression of NRGs in training set. (B). ROC curve of NRGs in training set. (C). ROC curve of NRGs in independent data set GSE104954. NRGs, NETosis-related genes.
