# Supplementary material for: Identification and validation of immune-associated NETosis subtypes and biomarkers in anti-neutrophil cytoplasmic antibody associated glomerulonephritis
Source: Front Immunol. 2023 Jul 3;14:1177968. doi: 10.3389/fimmu.2023.1177968 (PMC10351423; doi:10.3389/fimmu.2023.1177968)
Supplement: Supplementary file 3 [file DataSheet_2.zip › Ethical approval/Ethical approval letter.pdf]

武汉大学中南医院医学伦理委员会科研项目伦理审批件

Ethical Approval for Clinical/Scientific Research Projects under Medical Ethics Committee, Zhongnan Hospital of Wuhan University

批件号：临研伦[2022122K]

<table>
<tr><td>项目名称
Project Name</td><td colspan="4">ANCA 相关性血管炎伴肾损害中 NETosis 模式和免疫特征的综合分析</td></tr>
<tr><td>申办者
Sponsor</td><td colspan="4">NA</td></tr>
<tr><td>科室
Department</td><td>肾内科</td><td colspan="2">主要研究者
Primary Investigator</td><td>高苹</td></tr>
<tr><td>项目来源
Project Resource</td><td colspan="4">研究者发起项目</td></tr>
<tr><td>审核清单
The List of Reviewed Documents</td><td colspan="4">1. 伦理委员会审查申请表
2. 研究者简历
3. 研究方案（版本号：V1.0，2019 年 12 月 12 日）
4. 知情豁免申请</td></tr>
<tr><td>审查类型
review Category</td><td>☑初始审查 Primary　☐修正审查 Amendment</td><td>审批方式
Review Type</td><td colspan="2">☐会议审查 Conference　☑快速审查 Quick</td></tr>
<tr><td>日期 Date</td><td>2022-08-02</td><td>地点 Place</td><td colspan="2">NA</td></tr>
<tr><td>主审委员
Chief judge member</td><td colspan="4">☑同意　☐修改后同意　☐修改后再审　☐不同意
主审委员：[signature] [signature]</td></tr>
<tr><td>审查意见
Review Opinion</td><td colspan="4">1. ☑同意　☐必要修改后同意　☐不同意
2. 论文发表请按照我院科研处的相关程序报批。
3. 伦理批件有效期：12 个月 。

主任委员或副主任委员或授权人签字：[signature]
Signature of Chairman or Vice Chairman: [signature]
武汉大学中南医院医学伦理委员会（盖章）
Medical Ethics Committee
Zhongnan Hospital of Wuhan University（Stamp）
[Stamp: 武汉大学中南医院医学伦理委员会]
日期 Date: 2022-08-02</td></tr>
<tr><td colspan="5">注意事项 Notice：</td></tr>
</table>

1. 请遵循 NMPA/GCP 原则和《赫尔辛基宣言》、遵循本伦理委员会批准的方案开展临床研究，保护受试者的健康与权利。

Please comply with NMPA / GCP Principles and Helsinki Declaration, and carry out clinical trials according to code of ethics approved by our medical ethics committee in protection of the health and rights of human subjects

2.研究过程中，对研究方案和知情同意书等相关文件的修改，均须得到伦理委员会审查同意后方可实施。

All paperwork modification related to research proposal and informed consent during the course of study are subject to approval of the Ethics Committee.

3.发生严重不良事件或影响研究风险受益比的非预期不良事件、违背方案、暂停/终止研究须及时报告本伦理委员会。

Report timely to the medical ethics committee on incidents with serious adverse impact, unexpected incidents negatively affecting risk benefit ratio, breach of program or suspension / termination of study.

4.根据项目来源类别提交跟踪审查材料。 Submit follow-up review paperwork based on project resource categories

5.凡是涉及人类遗传资源出口或按照国家规定必须经有关部门审批的内容，均需在项目执行前向有关部门申报并获得批准。非以产品注册为目的临床研究需在医学研究登记备案信息系统(：http://114.255.48.20)注册后方可研究。

All contents regarding exporting of human genetic resources or approval by responsive Departments of Government, should be applied and approved in advance.Clinical research not for product registration needs to be registered in the Medical Research Registration Filing Information System (http://114.255.48.20) before research.

伦理委员会声明 Statement：

本伦理委员会严格按照中国 GCP 及相关法律法规组成及工作。

The Ethics Committee is well organized and works strictly in accordance with the Chinese GCP and related laws and regulations.

伦理委员会地址：湖北省武汉市武昌区东湖路 169 号门诊楼 11 楼

Address: Outpatient building, 11 floor, No.169 Donghu Road, Wuchang District, Wuhan, Hubei, China

邮编 Post Code：430071；　　电话 Phone：+86-27-67812787
